# *Rps27a* might act as a controller of microglia activation in triggering neurodegenerative diseases

**DOI:** 10.1371/journal.pone.0239219

**Published:** 2020-09-17

**Authors:** Nasibeh Khayer, Mehdi Mirzaie, Sayed-Amir Marashi, Maryam Jalessi

**Affiliations:** 1 Skull Base Research Center, The Five Senses Health Institute, Iran University of Medical Sciences, Tehran, Iran; 2 Department of Applied Mathematics, Faculty of Mathematical Sciences, Tarbiat Modares University, Tehran, Iran; 3 Department of Biotechnology, College of Science, University of Tehran, Tehran, Iran; Southern Methodist University, UNITED STATES

## Abstract

Neurodegenerative diseases (NDDs) are increasing serious menaces to human health in the recent years. Despite exhibiting different clinical phenotypes and selective neuronal loss, there are certain common features in these disorders, suggesting the presence of commonly dysregulated pathways. Identifying causal genes and dysregulated pathways can be helpful in providing effective treatment in these diseases. Interestingly, in spite of the considerable researches on NDDs, to the best of our knowledge, no dysregulated genes and/or pathways were reported in common across all the major NDDs so far. In this study, for the first time, we have applied the three-way interaction model, as an approach to unravel sophisticated gene interactions, to trace switch genes and significant pathways that are involved in six major NDDs. Subsequently, a gene regulatory network was constructed to investigate the regulatory communication of statistically significant triplets. Finally, KEGG pathway enrichment analysis was applied to find possible common pathways. Because of the central role of neuroinflammation and immune system responses in both pathogenic and protective mechanisms in the NDDs, we focused on immune genes in this study. Our results suggest that "cytokine-cytokine receptor interaction" pathway is enriched in all of the studied NDDs, while "osteoclast differentiation" and "natural killer cell mediated cytotoxicity" pathways are enriched in five of the NDDs each. The results of this study indicate that three pathways that include "osteoclast differentiation", "natural killer cell mediated cytotoxicity" and "cytokine-cytokine receptor interaction" are common in five, five and six NDDs, respectively. Additionally, our analysis showed that *Rps27a* as a switch gene, together with the gene pair {*Il-18*, *Cx3cl1*} form a statistically significant and biologically relevant triplet in the major NDDs. More specifically, we suggested that *Cx3cl1* might act as a potential upstream regulator of *Il-18* in microglia activation, and in turn, might be controlled with *Rps27a* in triggering NDDs.

## 1 Introduction

Neurodegenerative diseases (NDDs) are increasing serious menaces to human health in recent years [1]. In spite of the considerable body of research on NDDs such as Alzheimer’s disease (AD), amyotrophic lateral sclerosis (ALS), Huntington’s disease (HD), multiple sclerosis (MS), Parkinson’s disease (PD) and schizophrenia (SCHIZ), effective treatment for preventing the progressive neuronal loss is yet to be available [2].

Despite exhibiting different clinical phenotypes and selective neuronal loss in neurodegenerative abnormalities, there are many common pathological features that contribute to these disorders, which suggest the existence of certain common pathways at the molecular level. A few of such common pathological features are oxidative stress [3], mitochondrial dysfunction [4], glutamate excitotoxicity [5], abnormal protein aggregation [6], abnormal cellular transport [7], iron accumulation [8] and neuroinflammation [9, 10]. Therefore, it would be interesting to survey the NDDs at once to find such a potential similarity at the molecular level.

The number of comparative studies in NDDs is still rather low. Therefore, our knowledge is limited to the underling common pathways and central genes in these disorders. In a few previous studies, the major NDDs were comparatively surveyed at the systemic level. Durrenberger and coworker generated comparative genome-wide gene expression data for six main NDDs, namely AD, ALS, HD, MS, PD, and SCHIZ. They performed whole-genome expression analysis to look for potential common molecular pathogenic mechanisms and genes, in addition to exclusive disease-specific changes. They reported that no dysregulated gene was found in common across all six NDDs. Furthermore, they reported that "neuronal homeostatic", "survival activity" and "synaptic plasticity" pathways are shared among five and four of the diseases, respectively [11]. On the other hand, Godini and coworkers performed a comparative system-level analysis on NDDs microarray data that was generated by Durrenberger and coworkers. They constructed a protein-protein interaction network based on the co-expression approach and performed gene set enrichment analysis to find out the central factors that are related to each disease and, in addition, those features that can be considered as the similarities among them. Finally, they suggested a set of key genes that might contribute to all NDDs. Nevertheless, in this study, similar to the above mentioned, no pathway or gene was found to be in common across all six major NDDs [12].

**Fig 1:**
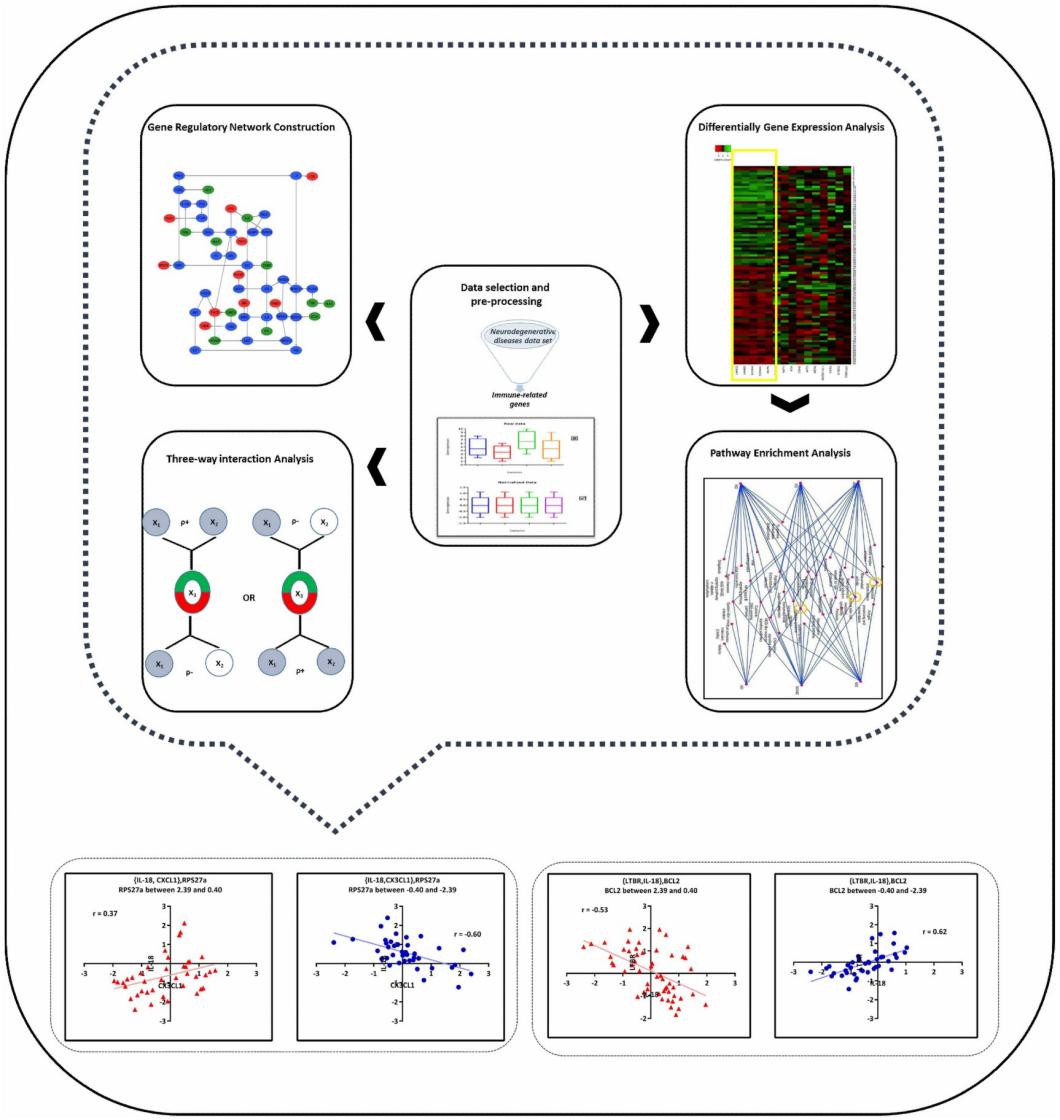
Schematic representation of the gene expression data analysis pipeline.

In the present work, we applied the three-way interaction model to find possibly shared genes and pathways among the major NDDs (Fig 1). High-throughput gene expression microarray data is a form of genome-scale data that provide an opportunity to trace gene expression patterns. There are numerous gene expression patterns which contain diverse information about gene relations [13, 14]. One of them is cross-shaped co-expression pattern. Such pattern is observed when the expression levels of two genes are either directly or inversely correlated depending on the expression level (or genotype) of a third gene (i.e., the switch gene). Three-way interaction mechanism can be inferred from a cross-shaped co-expression pattern. In this model, the changing nature of co-expression relationship of two genes{X_1_, X_2_} is captured by introducing a third gene (X_3_) which modulates the X_1_-X_2_ expression dependence. Therefore, such model can potentially shed light on some sophisticated gene interactions that cannot be traced trace with the simplistic correlative model. In addition, three-way interaction model can provide valuable information about gene regulatory relations [13, 15].

A significant body of evidence shows that neuroinflammation and immune system responses have both pathogenic and protective roles in the neurological disorders [9, 16–18]. Microglia and other innate immune cells such as astrocytes with a morphologically activated phenotype are observed at relatively early stages in large numbers of NDDs. Such cells can be activated in response to misfolded proteins, aberrantly localized nucleic acids or reactive oxygen species (ROS) [19]. Activation of innate immune cells leads to sever inflammatory responses in the central nervous system (CNS), renewed promoting neural damage. More specifically, Toll-like receptors (TLRs) and other pattern recognition receptors, which are expressed on microglia, play central roles in triggering neuroinflammation. Neural damage and subsequent inflammatory responses result in a feed-forward loop of neurodegenaration [19].

In the present study, we focus on the immune genes to look for NDDs related mechanisms and central switch genes that are involved in the immune responses using the three-way interaction model.

## 2 Materials and methods

### 2.1 Dataset selection and analysis

In the present work, we exploited the gene expression data reported by Durrenberger et al. [11]. The selected dataset includes comparative genome-wide gene expression data for AD, ALS, HD, MS, PD and SCHIZ from 118 cases of postmortem brain tissues, belonging to entorhinal cortex, cervical spinal cord, ventral head of the caudate nucleus, substantia nigra, grey matter in brodmann area, respectively. The data were generated using the Illumina humanRef-8 v2.0 expression bead chip platform and are available in GEO dataset under accession number GSE26927 [11]. A brief description of the dataset was presented in S1 Table in S1 Material. The duplicated probes were removed using the genefilter package in Bioconductor [20]. For this purpose, the mean of expression levels of duplicated probes were retained for each gene. Finally, the probes with "immune" annotations were selected based on the Biological Processes of the Gene Ontology annotation for the rest analysis [21].

Additionally, in order to identify differentially expressed genes (DEGs), for each disease, theempirical Bayes t-test was performed using the LmFit function in the Limma package [22] for each disease separately.

### 2.2 Determining liquid association triplets

Three-way interactions among all passible triplet combinations of genes in the dataset were calculated using fastMLA function in fast Liquid Association package [23]. This package uses a modified liquid association algorithm for determining changes in co-expression relations of two genes, X_1_ and X_2_, based on the expression level of a third gene (X_3_).

Before running liquid association algorithm, performing two preprocessing steps on the data is necessary: (i) normal quntile transformation [24]; (ii) standardization of mean and standard deviation to o and 1, respectively [25] for each variable. The first preprocess was performedby an in-house implementation and the second one by using CTT package [26].

False discovery rate (FDR) was estimated by using the Bonferroni correction method [27]. Subsequently, the three-way interactions with FDR < 0.01 were reported as the statistically significant triplets.

### 2.3 Construction of gene regulatory network

A gene regulatory network (GRN) model provides a systematic knowledge about the complex molecular mechanisms that control gene expression under diverse biological conditions [28]. Here, we used geWorkbench_2.6.0 software to construct GRN among all genes in dataset as hub markers by considering p-value <0.05. Specifically, ARACNE (Algorithm for the Reconstruction of Accurate Cellular Networks) was applied [29] to construct the GRN. This algorithm uses mutual information as a measure to detect direct regulatory interactions between each transcriptional regulator and its potential target(s).

### 2.4 KEGG pathway enrichment analysis

Kyoto Encyclopedia of Genes and Genomes (KEGG) pathway enrichment analysis is a popular statistical method to determine the validation of the shared association of a set of genes using predefined pathways [30]. Here, KEGG pathway enrichment analysis was performed separately for each of the six sets of DEGs using the KEGG database [31]. Above-mentioned analyses were performed using ClueGO plugin [32] within the Cytoscape v.3.3.0 environment [33], and in addition, two-sided hypergeometric test with the Bonferroni step down correction method and a kappa score threshold of 0.4 were considered for statistical significance calculation.

### 2.5 Ethics statements

The presented study is a computational research using a public GEO dataset (accession number: GSE26927). Therefore, any ethics approval and consent statements were not required for this study.

## 3 Results

### 3.1 Preprocessing dataset and identifying differentially-expressed genes

After preprocessing and extracting the immune-related genes from dataset, 822 genes remained for further analysis. In order to determine differentially-expressed genes, eBayes t-test was performed on the normalized data. By considering *p*-value <0.05, we selected 21, 150, 171, 111, 145 and 83 genes as significant differentially-expressed genes in AD, ALS, HD, MS, PD and SCHIZ, respectively. The detailed results of this analysis are available in S2 Table in S1 Material.

### 3.2 Determining the significant cross-shaped triplets

**Fig 2:**
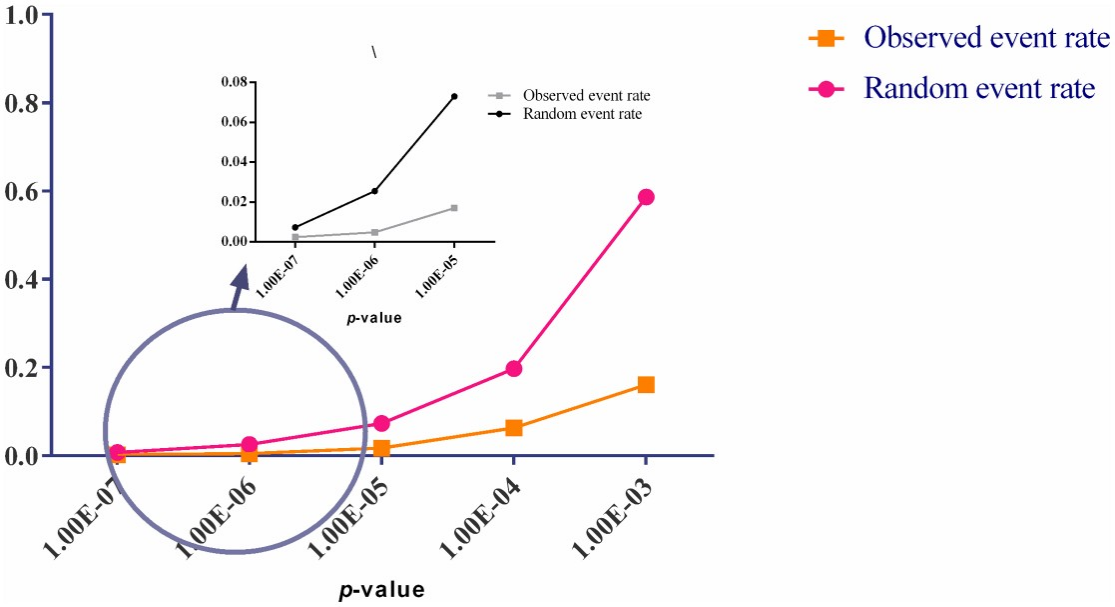
A survey of validity of fastLA analysis. In the wide range of the significant fastLA *p*-values, observed event rate of X_3_ position (switch) genes were compared with random event rate. As shown, the observed event rate for switch genes is far greater than the random one, which confirms the validity of fastLA analysis.

**Fig 3:**
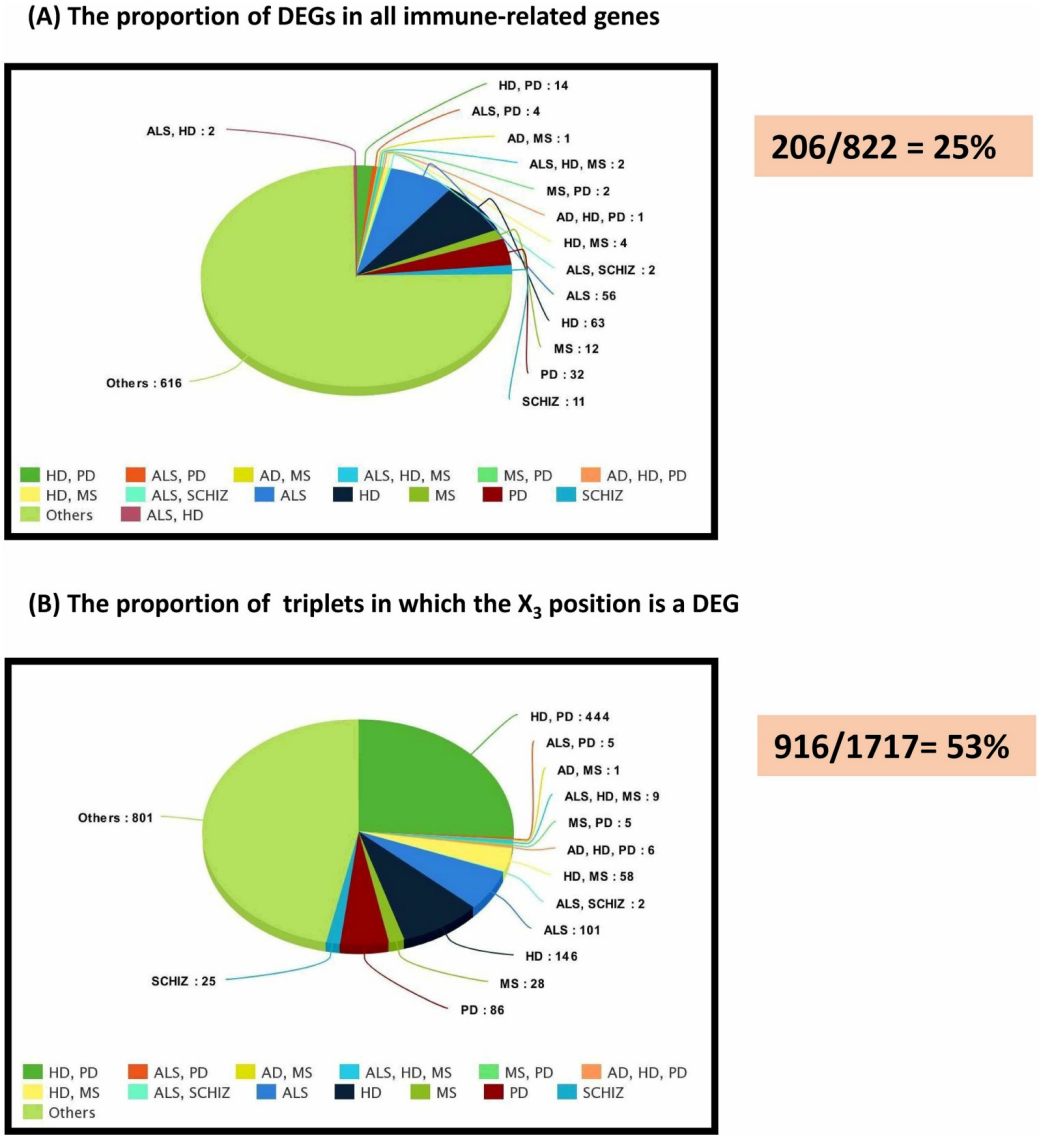
Relevancy of switch genes with Differentially Expressed Genes (DEGs). (A) The proportion of DEGs in all immune-related genes; and (B) The proportion of triplets in which the X_3_ position is a DEG. As shown, the percentage of DEGs in A and B is 25% and 53%, respectively. The prevalence of DEGs in the X_3_ position of triplets suggest that the liquid association model is appropriate for understanding the role of DEGs in neuroinflammation.

All 822 immune-related genes were considered as potential switch genes in the fast liquid association analysis. The changes in FDR versus -log(*p*-value) for the first 300000 triplets were showed in S3 Fig in S1 Material. In order to survey the accuracy of fastLA analysis, observed event rate of X_3_ position (switch) genes in the wide range of the significant fastLA *p*-values were compared with random event rate (see Fig 2). On the other hand, the relevancy of X_3_ position (switch) genes with DEGs was examined. As shown in Fig 3, the percentage of the DEGs in all of the immune-related genes and the percentage of triplets that X_3_ position of them is DEG in all statistically significant triplets is 25% and 53%, respectively.

With considering FDR <10^−2^, a set of significant cross-shaped triplets which consists of 1717 triplet combinations were selected for further analyses.

### 3.3 Identifying common KEEG pathway(s) in NDDs

**Fig 4:**
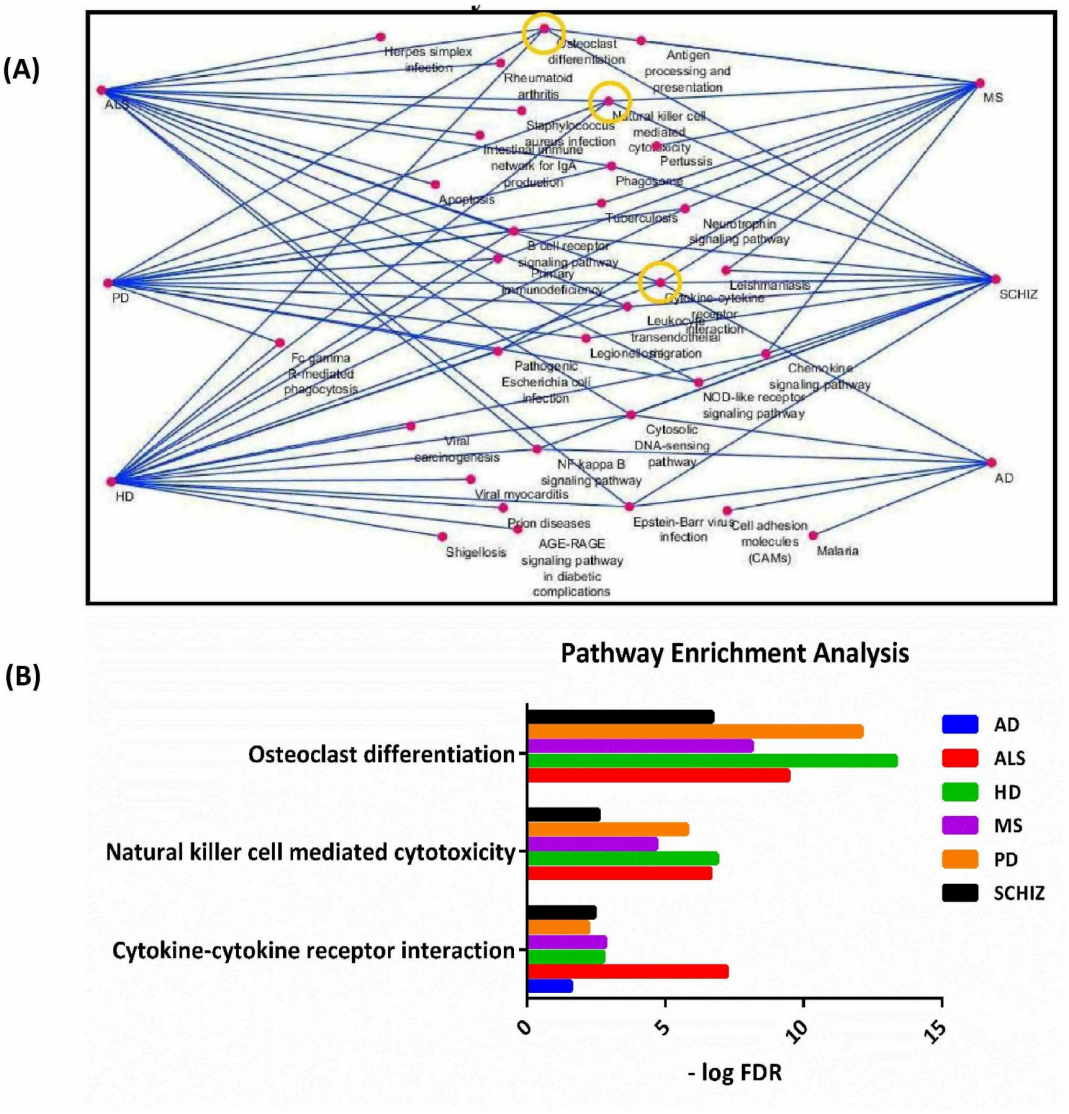
Pathway enrichment analysis. Enriched terms based on KEGG pathway (A) for each neurodegenerative disease (NDD) separately; and (B) shared by at least five of the NDDs.

We used GSEA in order to find common pathway(s) in NDDs in addition to detect the biologically-relevant triplets. By considering FDR <0.05 and a minimum of 3 genes in each groups, three pathways were enriched simultaneously at least in 5 of the NDDs. All of the significantly enriched terms based on "KEGG pathway" were presented in Fig 4A, and in addition, a detailed information about KEGG pathway enrichment analysis was reported in S4 Table in S1 Material. Based on the results, "cytokine-cytokine receptor interaction" was enriched in all 6 NDDs, while "osteoclast differentiation", “natural killer cell mediated cytotoxicity" were enriched in 5 of the NDDs each (Fig 4B). Based on the definition of three-way interactions in switch mechanism model, it is expected that in biologically-relevant triplets, X_1_ and X_2_ are in the same pathway. By detecting the statistically significant triplets in the enriched terms, 89 triplets were found to be biologically relevant based on GSEA. These triplets are reported in S5 Table in S1 Material.

### 3.4 Detecting the cross-shaped triplets in GRN

As another attempt for analyzing the functional relevance of statistically significant triplets, we reconstructed a GRN by ARACNE. By considering *p*-value <0.05, the GRN included 3199 edges and 822 nodes. Consequently, the statistically significant triplets were mapped on to the GRN. The results showed that the regulatory relations of 242 triplets are detectable in this GRN.

**Fig 5:**
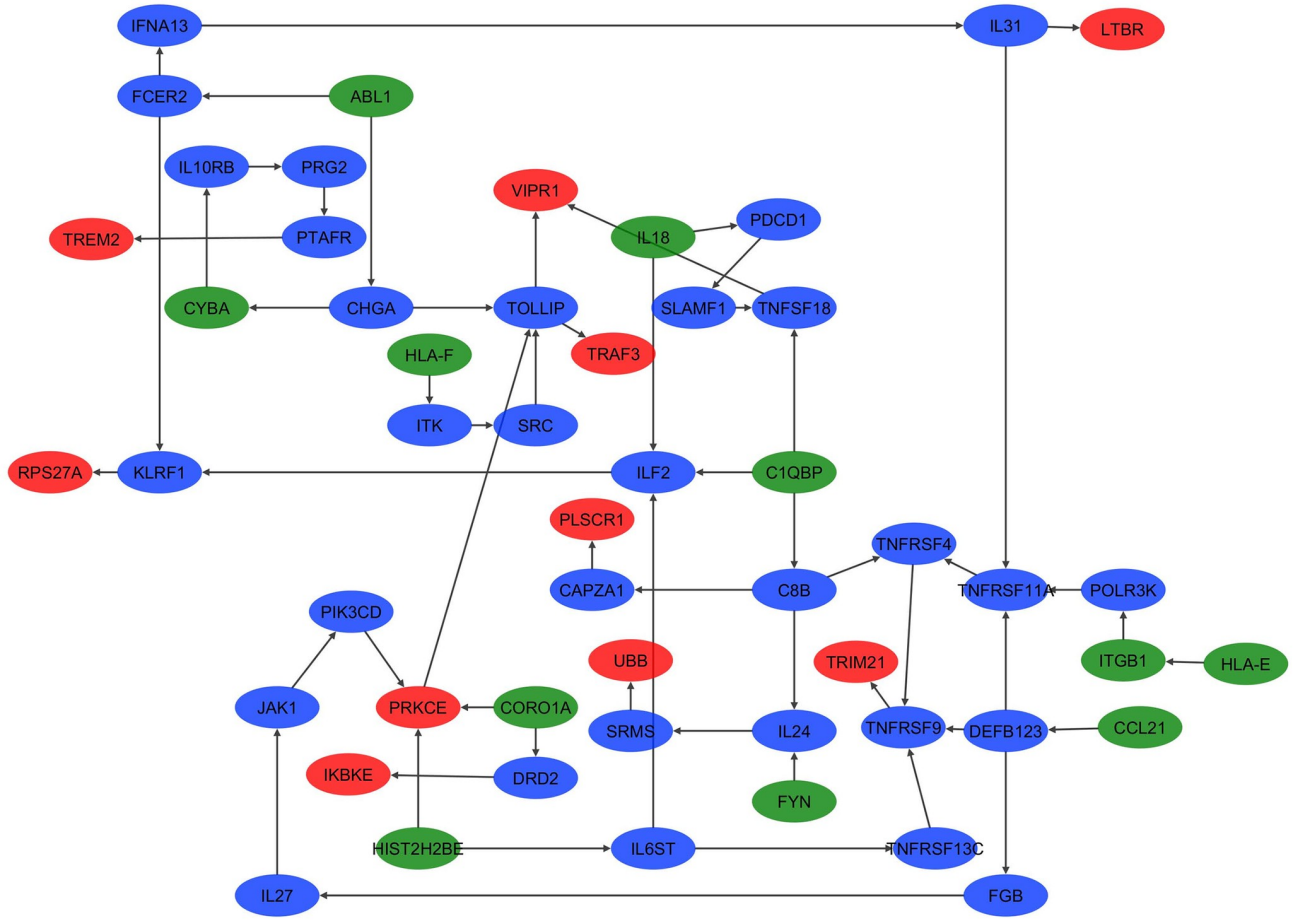
The position of biologically relevant triplets in Gene Regulatory Network (GRN). The biological relevance of 14 statistically significant triplets was confirmed by KEGG pathway enrichment analysis as well as GRN analysis. A subnetwork of GRN that includes the regulatory relations of such triplets is shown here. Red nodes represent the X_3_ position gene in each triplet, green nodes represent the X_1_ and X_2_ position genes, and other genes are presented by blue nodes.

**Fig 6:**
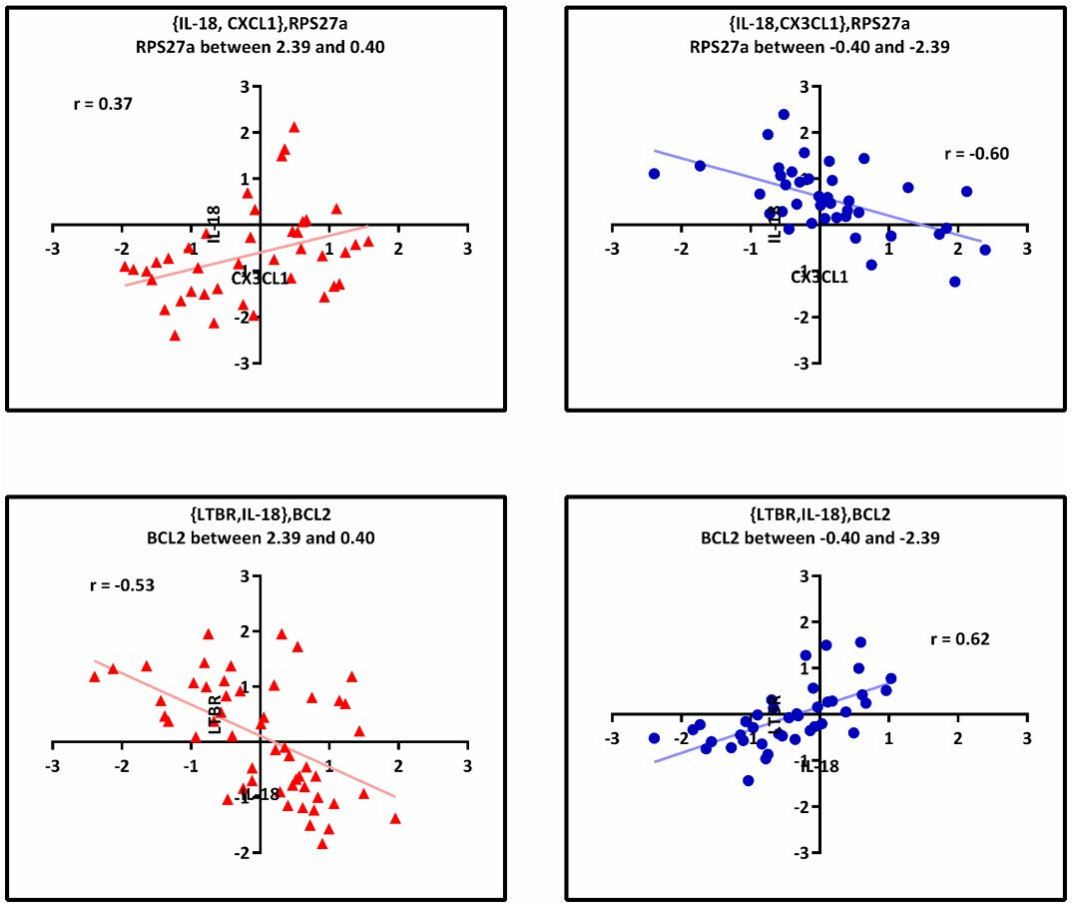
Scatter plot of the two statistically significant and biologically relevant triplets. In each case, there is a considerable change in the correlation of X_1_ and X_2_ as a result of change in X_3_ expression level.

Overall, the biological relevance of the 14 statistically significant triplets was defined by both GRN and GSEA methods. The position of such triplets are shown as a subnetwork of GRN in Fig 5. In addition, the liquid association analysis information of these triplets were reported in Table 1. One of these triplets is the 1133^nd^ triplet, which consists of genes *Rps27a* and {*Il18*, *Cx3cl1*}. Although some triplets have a higher liquid association score than 1133^nd^ triplet, there is a feature that distinguishes such triplets from the other ones. 1133^nd^ triplet is the only statistically significant triplet that is not only biologically relevant based on both GRN and GSEA analyses but also it is involved in 5 diseases (i.e., across all six NDDs except ALS). On the other hand, the biological relevance of 1328^nd^ triplet, which is involved in common across all 6 NDDs, was confirmed only by GSEA method. This triplets consists of genes *Bcl2* and {*Ltbr*, *Il18*}. The scatter plots of 1133^nd^ and 1328^nd^ triplets in three different ranges of expression level of their associated X_3_ are presented in Fig 6. As shown, a considerable change in the correlation of X_1_ and X_2_ occurs as a result of the changes in X_3_ expression level.

**Table 1:** Biologically relevant triplets.

X1 or X2	X2 or X1	X3	ρ_high_	ρ_low_	ρ_diff_	*p*-value	adjusted *p*-value
*Coro1a*	*Cyba*	*Ikbke*	0.77 ↑↑	-0.58 ↑↓	1.35	4.30E-11	8.60E-06
*Traf3*	*Cyba*	*Trem2*	-0.11 ↑↓	-0.67 ↑↓	0.56	8.09E-10	1.62E-04
*Ticam1*	*Il18*	*Vipr1*	-0.4 ↑↓	0.77 ↑↑	-1.17	1.73E-08	3.46E-03
*Itgb1*	*Coro1a*	*Ikbke*	0.51 ↑↑	-0.64 ↑↓	1.15	1.89E-08	3.78E-03
*Tnfrsf1a*	*Coro1a*	*Ikbke*	0.65 ↑↑	-0.59 ↑↓	1.24	2.31E-08	4.62E-03
*Il18*	*Cx3cl1*	*Rps27a*	0.37 ↑↑	-0.56 ↑↓	0.93	2.39E-08	4.78E-03
*Abl1*	*Hla-F*	*Traf3*	0.6 ↑↑	-0.27 ↑↓	0.87	2.47E-08	4.94E-03
*Tlr4*	*Abl1*	*Ltbr*	-0.12 ↑↓	0.73 ↑↑	-0.85	2.90E-08	5.80E-03
*Hla-E*	*Coro1a*	*Ikbke*	0.6 ↑↑	-0.58 ↑↓	1.18	2.99E-08	5.98E-03
*Ticam1*	*C1qbp*	*Plscr1*	-0.6 ↑↓	0.39 ↑↑	-0.99	3.07E-08	6.14E-03
*Nfkb1*	*Coro1a*	*Ikbke*	0.57 ↑↑	-0.66 ↑↓	1.23	3.21E-08	6.42E-03
*Hla-E*	*Hist2h2be*	*Trim21*	-0.38 ↑↓	0.53 ↑↑	-0.91	3.33E-08	6.66E-03
*Fyn*	*Tnfrsf10b*	*Ubb*	0.64 ↑↑	-0.56 ↑↓	1.20	3.53E-08	7.06E-03
*Ccl21*	*Il18*	*Prkce*	0.57 ↑↑	-0.66 ↑↓	1.23	4.25E-08	8.50E-03

The liquid association analysis information of the 14 triplets that are statistically, as well as biologically, significant. The Pearson’s correlation coefficient of X_1_ and X_2_ when X_3_ is at a high gene expression level is indicated by ρ_high_ (i.e., rho{X_1_, X_2_}|X_3_↑) and when X_3_ is at a low gene expression level is indicated by ρ_low_ (i.e., rho{X_1_, X_2_}|X_3_↑). In addition, ρ_diff_ is the absolute value of their difference (i.e., ρ_diff_ = |ρ_high-_ρ_low_|). Arrows indicate direct (↑↑) or inverse (↑↓) correlation of X_1_ and X_2_ in the specific state of X_3_.

## 4 Discussion

Although different NDDs are clinically characterized with certain distinctive features, these diseases exhibit share common neuropathological characteristics [34]. One such characteristic is neuroinflammation which is associated with the immune system processes such as inflammation and immune response. Previously, a number of studies investigated the molecular factor(s) of such common changes [11, 12]. However, to the best of our knowledge, no pathway or gene was reported to be in common across all major NDDs. In the present study, the three-way interaction model was used for to investigate the immune-related switch mechanisms in NDDs. Our results suggests that a number of immune-related switch genes act variety of different NDDs.

As the mentioned above, the purpose of the present study was to perform a comparative analysis on the involved mechanisms in NDDs. Because distinct regions of brain are affected in different NDDs [35], there was an inevitable diversity among the brain regions from which the samples are taken. On the other hand, several studies show that the properties of cell population in samples depended on the region of brain [36]. Therefore, in order to reduce the variability between samples, a preprocessing and normalization step as the first stage was performed. More specifically, the quantile normalization method [37] was utilized to minimize the batch effects.

The results of eBayes t-test analysis suggest that the number of DEGs are strikingly different among these diseases. The lowest and the highest number of immune-related DEGs belong to AD and HD, respectively. This observation is consistent with the idea that beside a number of morphological similarities, fundamental differences exist between the natures of these diseases.

As shown in Fig 2, the observed event rate of X_3_ position (switch) genes in a wide range of significant fastLA *p*-values is far greater than what is expected by chance. In other words, most X_3_ positions in the statistically significant triplets are occupied by certain genes. Therefore such result confirms the validity of fastLA analysis. In addition, in Fig 3, we show that such certain X_3_ positions genes are frequently belong to DEGs. As shown in Fig 3, the percentage of the DEGs in the all of the immune genes is 25%, while the percentage of DEGs in the all of the X_3_ position genes is 53%. This results shows that DEGs play a central role in the switch mechanisms.

The results of the KEGG enrichment analysis (Fig 4) show that three pathways are commonly involved in at least five of the NDDs. These pathways include "osteoclast differentiation", "natural killer cell mediated cytotoxicity" and "cytokine-cytokine receptor interaction". In the following, the relationship between these pathways and NDDs is explained in detail.

### 4.1 Osteoclast differentiation

From its name, the osteoclast differentiation pathway seems to be an unrelated pathway to the NDDs at the first sight. It is interesting to note that the link between osteoclast differentiation pathway and the NDDs is suggested in numerous studies [38–43]. Previous studies reported that the oncostatin M receptor signaling pathway, which is associated with NDDs [44, 45], stimulates osteoclast differentiation [46, 47]. On the other hand, Sato and colleagues pointed out donepezil, a typical drug for Alzheimer’s disease [48], inhibits osteoclast differentiation by down regulating acetyl cholinesterase [49]. In addition, ROS are involved in Alzheimer’s disease as well as the osteoclasts differentiation pathway. Specifically, ROS, as redox signaling intercellular molecules, are involved in regulating the osteoclast differentiation via receptor activator of nuclear factor kappa β ligand. On the other hand, cytotoxic effect of ROS is associated with destruction of lipids, proteins and DNA in AD. In addition, previous studies reported that the vesicle-associated membrane protein (VAMP)-associated protein B (VAPB) [50–52], which is related to the osteoclast differentiation pathway, is involved in NDDs such as ALS [53], and PD [54]. In fact, VAPB participates in death of motor neurons through ER stress and impaired Ca^2+^ homeostasis [53]. Furthermore, VAPB regulates the osteoclast differentiation pathway by adjusting NFATc1 [55].

### 4.2 Natural killer cell mediated cytotoxicity

Another enriched biological pathway for NDDs is "Natural killer cell mediated cytotoxicity" (Fig 4). Natural killer (NK) cells are large granular lymphocytes that have cytotoxic properties [56]. The cytotoxicity of NK cells is carried out a collectively by inhibitory and stimulatory receptors that are expressed on NK cells surface [57]. In addition, NK cells have a crucial role in innate and adaptive immune system [58]. The central role of the NK cell activity in AD, MS, PD and SCHIZ has been reported (see below).

Some evidence suggests that the NK cell activity in AD patients is significantly lower than that in normal controls [59]. Moreover, the cytotoxic activity of NK cells is associated with neurological inflammation observed in neuronal degeneration [60]. In addition, the NK cells activity has been suggested as a biomarker for AD progression [61]. Likewise, the role of NK cell in MS disease has been well studied [56, 62]. Uchida and colleagues showed that NK cell activity in MS patients is significantly lower than that in normal ones. They also reported that the interferon treatment of lymphocytes leads to an increase in the activity of NK cells that is less than in MS patients compared to normal ones [63]. Chanvillard and colleagues showed that mitoxantrone, a cytotoxic drug that is approved for treatment of progressive MS, induces maturation of NK cells in patients with progressive MS [64]. Rodriguez-Martin and colleagues reported that all NK cell subsets significantly increased in MS and other inflammatory neurological diseases compared to in non-inflammatory neuropathies [65]. In addition, Segal reported that in MS, NK cells curb neuroinflammation by inhibiting the auto-reactive T cell responses. In other words, NK cells by regulating of some adaptive immune responses, restricts neuroinflammation in MS [66]. Furthermore, the role of NK cells in PD has been also studied. Mihara and colleagues reported that expression of inhibitory receptor CD94/NKG2A was remarkably lower in PD patients than those in non-PD individuals. Actually, inhibitory receptor CD94/NKG2A suppresses activation signaling processes, which trigger after binding NK cells with its ligand on the normal cells, to avoid destruction of normal bystander cells [67]. Therefore, because of limited number of such inhibitory receptor in PD patient and subsequently a deficiency in inhibition of NK cells activation in the normal cells, the PD patients are prone to disruption of normal cells.[68]. Jiang and colleagues conducted a systematic review to found relevance between lymphocyte subsets/NK cell and the risk of PD. Their results showed that the number of NK cells significantly increased in PD; and consequently, such cells may be associated with the risk of PD [69]. Additionally, there are numerous studies on the activation of NK cells in SCHIZ. Schindler and colleagues compared the activity of NK cells in the blood of SCHIZ patients and healthy controls, but they did not find significant differences between the two groups [70]. On the other hand, Abdeljaber and colleagues reported that the activity of NK cells in SCHIZ patients decline compared to the control group [71], although similar results are not observed in other studies.

### 4.3 Cytokine-cytokine receptor interaction

Another biological pathway that is enriched in the six major NDDs is "cytokine-cytokine receptor interaction" (Fig 4). Cytokines are produced by peripheral immunocompetent, glial cells and neurons [72]. During immune responses, cytokines mediate specific signaling communications between neurons and immune cells. Additionally, cytokines play pleiotropic roles in the central nervous system, which include roles in synaptic plasticity, neurotransmission, nuclear signal transduction, neurogenesis, and inflammatory responses [73, 74]. While the pathophysiological role of cytokines in NDDs is well known [75], to our knowledge, the cytokine-cytokine receptor interaction (CCRI) pathway was reported only in MS, SCHIZ, and AD in previous studies. See below.

Xu and colleagues have previously indicated that the change in of *Il-18* pathway may contribute to psychopathology of SCHIZ. They also showed that the *Il-18* pathway was strongly associated with the CCRI pathway [74]. The relevance of the pathway to MS has been also reported. Guo and colleagues, by comparison between blood transcriptome of MS and healthy individuals, showed that genes involved in the CCRI pathway were potentially associated with MS [76]. Likewise, about AD, Sattlecker and colleagues reported that the members of CCRI pathway change during AD in proteome-level quantification [77]. On the other hand, Khayer and colleagues reported that CCRI pathway is a critical switch pathway in triggering AD [15].

### 4.4 Relationship between involved genes in triplet *Rps27a*, {*Il-18*, *Cx3cl1*}

The 1133^nd^ triplet which contains of genes *Rps27a* and {*Il-18*, *Cx3cl1*} is one of the most statistically significant triplets in our analysis. Furthermore, this triplet is found to be biologically relevant, based on GSEA and GRN analyses (Table 1 and Fig 5). As shown in Fig 6, when normalized expression level of *Rps27a* gene is between 0.40 and 2.39, there is an inverse correlation between *Il-18* and *Cx3cl1* expression levels. In contrast, when normalized expression level of *Rps27a* gene is between -2.39 and -0.40, there is a direct correlation between *Il-18* and *Cx3cl1* expression levels. In other words, changes in expression level of *Rps27a* gene can act as the switch factor for altering the regulated genes.

To the best of our knowledge, no previous study has investigated the role of *Rps27a* gene in NDDs. Nevertheless, there is considerable evidence to support that *Rps27a* gene may be associated with this category of diseases.

*Rps27a* gene encodes a fusion protein consisting of ubiquitin (Ub) at the N terminus and ribosomal protein S27a at the C terminus [78]. Rps27*a* protein is cleaved to free ubiquitin monomer and ribosomal protein S27a by a cysteine protease, namely *UCHL1* [79]. In other words, *UCHL1* is a deubiquitinating enzymes (DUBs).

Ub is a versatile regulatory protein that plays diverse roles in cells [80]. In addition, the cellular Ub pool, which is composed of free Ub and Ub conjugates, are in dynamic equilibrium inside cells. On the other hand, the maintenance of cellular levels of Ub (i.e., Ub homeostasis) is substantial for appropriate ubiquitination of substrate proteins [81]. Previous studies indicated that disruption of Ub homeostasis plays a critical role in the pathophysiology of various NDDs [82–86]. There are at least two ways for maintaining Ub homeostasis in cells. One of them is regulation of DUBs that convert Ub conjugates into their monomeric free forms, and consequently increase the levels of Ub pool [87]. UCHL1, a DUB enzyme for *Rps27a* (see previous paragraph), dysfunction is associated with a variety of NDDs, including AD [88, 89], PD [90–92], HD [93, 94] and SD [95]. The other way is regulation of gene expression of monomeric Ub-ribosomal fusion genes as well as polyubiquitin genes. As mentioned above, *Rps27a* is a monomeric Ub-ribosomal fusion gene; therefore regulation of such gene can be a way to maintain cellular Ub pool and in turn, might contribute to NDDs.

As it was mentioned above, *Il-18* and *Cx3cl1* genes that are involved in triplet 1133^nd^ are directly correlated in some conditions. Surprisingly, the positive relationship between *Il-18* and *Cx3cl1* was reported in previous studies. Alboni and colleagues demonstrated that fluoxetine, as a chronic antidepressant drug, that is involved in anti-inflammatory, anti-apoptotic and antioxidant activity [96], decreases hypothalamic expression of *Il-8* and *Cx3cl1* genes simultaneously [97]. On the other hand, both *Il-18* and *Cx3cl1* genes were suggested as interferon-related biomarkers for *Listeria monocytogenes* by Koopmans and colleagues [98]. In addition, Kasama and colleagues reported that gene expression levels of *Cx3cl1* and *Il-18* are positively correlated with each other in adult-onset Still’s disease [99]. Furthermore, Bian and collegues indicated that the interaction between *Cx3cl1* and *Il-18* signaling plays a critical role in the development of allodynia [100].

### 4.5 *Cx3cl1* as a potential upstream regulator of *Il-18* in microglia activation

Some evidence suggests that *Cx3cl1* might be as an upstream regulator of *Il-18* in microglia cells. See below.

*Cx3cl1* and *Il-18* genes are involved in spinal long term potentiation [101] as well as microglia activation [102]. Bian and colleagues, by utilizing double immunostaining technique, showed that *Cx3cl1* receptor (*Cx3cr1*) is co-localized with *Il-18* in the spinal cord and predominately expressed in spinal microglia in rats. They also reported that blockade of *Cx3cl1*/ *Cx3cr1* signaling and/or *Il-18*, obviously suppresses the spinal long term potentiation [101]. On the other hand, Miyoshi and colleagues determined that production of *Il-18* in the spinal cord was regulated by *p38MAPK* [103]. Furthermore, Zhang and colleagues reported that the p38MAPK signaling was activated in spinal microglia by exposing to exogenous *Cx3cl1* [104]. Therefore, it is reasonable to infer that *Cx3cl1* might act as a potential upstream regulator of *Il-18* in microglia cells.

Besides, some previous studies indicate that microglia activation is involved in spinal long term potentiation [105, 106] as well as in NDDs [107]. It should be mentioned that the central role of *Il-18* gene in AD [108], ALS [109, 110], MS [111] and SD [74, 112, 113], and *Cx3cl1* gene in all of the six surveyed diseases (i.e. AD [114, 115], ALS [116], HD [117], MS [118], PD [119–122] and SD [123, 124]) has been determined. However, as far as our knowledge goes, the relation between *Il-18* and *Cx3cl1* in triggering of NDDs is not reported in the literature yet.

### 4.6 Investigation of DAM-like and primed-like expression program during human neurodegeneration

Microglia, the central participator in the immune system of CNS, are versatile cells. Such cells, depending on environment signals, can acquire different phenotypes [125]. Two novel known subtypes of microglia that occur in mouse models of NDDs, are disease-associated microglia (DAM) [126] and primed microglia [127]. In this section, we asked whether induction of a DAM-like and/or primed-like expression program is traced in microglia of human NDDs. To this end, we compared our results with recently published gene expression data of DAM and primed microglia. See below.

Recently, Keren-Shaul and co-authors [126] reported a protective sub-type of microglia associated with NDDs, termed disease-associated microglia (DAM), in AD and ALS mouse model. They utilized single-cell RNA sequencing technique on microglia isolated from mouse model of AD (5XFAD) and ALS (mSOD1 [G93A] mice) as well as the corresponding wild-type mice. Subsequently, using graph-based clustering analysis, they identified a DAM gene signature for mouse model of AD and ALS. We compared our results with DAM characteristic genes to investigate the DAM-like expression program in human NDDs. Of the 22 DAM characteristic genes, reported by Keren-Shaul and co-authors, 7 genes, including *Cx3cr1*, *Tyrobp*, *B2m*, *Fth1*, *Trem2*, *Axl* and *Csf1*, were found in our results as involved genes in statistically significant triplets. Additionally, *Trem2*, a key gene that enable the microglial progression to a full activation DAM profile, was found in our results as a switch gene. On the other hand, they highlighted lysosomal, phagocytic, and lipid metabolism pathways for DAM characteristic genes. In a comparative aspect, none of DAM-related pathways were enriched in our study for any disease. Taken together, there are not a striking overlap between DAM characteristic genes and key genes reported in our study. Two possible reasons for such observation may be: (i) differences in applied transcriptomic analysis techniques, which can affect the accuracy of the study; (ii) differences in the nature of investigated samples. To the best of our knowledge, a slight genetic differences between mouse and human might be translated in to a salient distinction [128].

Another subtype of microglia that is reported for mouse model of NDDs is primed microglia. Such subtype of microglia acquires a hypersensitive phenotype, becoming hyper-reactive and secreting large amount of inflammatory mediators in response to signal from a systemic inflammatory event [129]. Holtman and co-authors [127], to determine the gene expression signature of priming, analyzed the transcriptome of microglia in mouse model of AD and ALS using weighted gene co-expression network analysis. They identified four hub genes specific for the primed microglia expression network, including *Lgals3*, *Igf1*, *Csf1*, and *Axl*. Interestingly, all of hub genes except *Igf1* were found in our results. These genes are involved in X_3_ position and/or X_1_, X_2_ of significant triplets. Hence, it can be expected that a primed-like expression program triggers in human neurodegenerative diseases as in mouse model occurs. However, our knowledge for such claim is not sufficient and more evidence is needed to support it.

## 5 Conclusion

Recent study is the first time that was utilized three-way interaction approach to survey common pathways in the NDDs. Three-way interaction approach is a promising strategy that provides new type of information on the specific relationships among genes. In conclusion, three common switch pathways that include "osteoclast differentiation", "natural killer cell mediated cytotoxicity" and "cytokine-cytokine receptor interaction" were found in the major NDDs. More specifically, our analysis showed that *Rps27a* as the switch gene and the gene pair {*Il-18*, *Cx3cl1*} form a statistically significant and biologically relevant triplet in the major NDDs. Additionally, we suggested that *Cx3cl1* might act as a potential upstream regulator of *Il-18* in microglia activation, and in turn, might be controlled with *Rps27a* in triggering NDDs.

## Supporting information

S1 Materials (PDF)

## Abbreviations

AD: Alzheimer’s disease
ALS: Amyotrophic Lateral Sclerosis
DEG: Differentially Expressed Gene
GRN: Gene Regulatory Network
GSEA: Gene Set Enrichment Analysis
HD: Huntington’s Disease
MS: Multiple Sclerosis
NDD: Neurodegenerative Disease
PD: Parkinson’s Disease
ROS: Reactive Oxygen Species
SCHIZ: Schizophrenia
