# Supplementary material for: Rps27a might act as a controller of microglia activation in triggering neurodegenerative diseases
Source: PLoS One. 2020 Sep 17;15(9):e0239219. doi: 10.1371/journal.pone.0239219 (PMC7498011; doi:10.1371/journal.pone.0239219)
Supplement: S1 Materials — (PDF) [file pone.0239219.s001.pdf]

PS1 Table. A brief description of dataset. Accession number: GSE26927; and platform: GPL 6255 Illumina humanRef-8 v2.0.

Neurodegenerative Diseases	Num of control Sample	Num of disease Sample	Brain Area
Alzheimer's Disease (AD)	7	11	Entorhinal Cortex
Amyotrophic Lateral Sclerosis (ALS)	10	10	Cervical Spinal Cord
Huntington's Disease (HD)	10	10	Ventral Head of the Caudate Nucleus
Multiple Sclerosis (MS)	10	10	Subpial Grey Matter Lesions in the Frontal Gyri
Parkinson's Disease (PD)	8	12	Substantia Nigra
Schizophrenia (SCHIZ)	10	10	Grey Matter in Brodmann Area

S2 Table. Significant differentially-expressed genes.

Gene name	logFC	AveExpr	t	P.Value	adj.P.Val	B	Disease
ADAR	-274.9152	2438.1234	-3.524552	0.0026636	0.9788947	-4.59415	AD
TNFSF12	-90.58247	456.94703	-3.347166	0.0038996	0.9788947	-4.594215	AD
BCL2	-65.65414	334.03547	-2.870278	0.0107552	0.9788947	-4.594402	AD
SPON2	-13.00418	45.585	-2.810461	0.0121932	0.9788947	-4.594426	AD
DEFB119	4.9940519	13.211222	2.7923578	0.0126639	0.9788947	-4.594433	AD
ENPP1	-17.00491	45.453889	-2.778807	0.0130276	0.9788947	-4.594439	AD
FADD	-54.27831	423.48122	-2.660289	0.0166674	0.9788947	-4.594488	AD
SEMA7A	12.718338	18.735111	2.6264681	0.0178734	0.9788947	-4.594502	AD
TREML2	9.3583766	27.717278	2.6097526	0.0184997	0.9788947	-4.594508	AD
CHUK	57.413494	557.11383	2.4710033	0.0245686	0.9788947	-4.594566	AD
HRG	-12.61374	33.172111	-2.455966	0.0253293	0.9788947	-4.594572	AD
MBP	967.74974	3025.8897	2.3250356	0.0329566	0.9788947	-4.594627	AD
MS4A2	-8.196909	50.607222	-2.240479	0.038971	0.9788947	-4.594662	AD
TRIM6	-5.218416	15.810889	-2.201735	0.0420539	0.9788947	-4.594678	AD
CCL5	14.180351	65.533944	2.1956148	0.042561	0.9788947	-4.594681	AD

CCR5	-6.565727	18.279389	-2.184166	0.0435247	0.9788947	-4.594685	AD
MIF	960.97001	4710.7838	2.1802054	0.0438628	0.9788947	-4.594687	AD
COCH	128.68123	366.26861	2.1705473	0.0446974	0.9788947	-4.594691	AD
COLEC12	-151.2906	362.985	-2.147134	0.0467815	0.9788947	-4.594701	AD
CXCL6	-8.442584	25.3685	-2.140755	0.0473646	0.9788947	-4.594703	AD
DEFB127	-8.02187	22.706111	-2.133182	0.0480655	0.9788947	-4.594707	AD
LILRB3	-1209.547	1154.3179	-6.068168	8.41E-06	0.005069	-2.953094	ALS
LTB4R	-184.0902	295.3052	-5.885916	1.23E-05	0.005069	-2.993571	ALS
TNFSF13B	-219.278	266.4962	-5.252921	4.81E-05	0.0131714	-3.14915	ALS
IL6R	-82.36345	115.62438	-5.110498	6.57E-05	0.0134979	-3.18754	ALS
HLA-E	-2298.124	5932.3501	-4.757669	0.0001435	0.0235929	-3.28826	ALS
MALT1	-115.7214	277.0673	-4.543881	0.0002316	0.0317224	-3.353241	ALS
TSC1	43.56565	213.27353	4.3965094	0.0003225	0.0378765	-3.399779	ALS
CD48	-267.9697	219.25565	-4.171967	0.0005355	0.0550241	-3.473393	ALS
TNFRSF1B	-1769.698	2991.9062	-3.866533	0.0010693	0.0732949	-3.578615	ALS
SP2	87.2189	348.89155	3.8401256	0.0011352	0.0732949	-3.587976	ALS
ITK	-32.181	54.3515	-3.792871	0.0012635	0.0732949	-3.604829	ALS
POLR3B	116.1395	541.33955	3.7598441	0.0013615	0.0732949	-3.616682	ALS
UBB	2347.4409	7311.5991	3.7523238	0.0013849	0.0732949	-3.61939	ALS
BCL6	-1212.928	2523.5615	-3.742943	0.0014146	0.0732949	-3.622772	ALS
CCL2	-1009.349	993.4117	-3.736056	0.0014368	0.0732949	-3.625258	ALS
CD226	9.8311	18.06895	3.664996	0.0016874	0.0732949	-3.651063	ALS
PAG1	-66.5334	116.2856	-3.644439	0.0017676	0.0732949	-3.658579	ALS
LCP2	-367.5695	693.64775	-3.631949	0.0018182	0.0732949	-3.663156	ALS
CD3G	-18.9636	32.849	-3.614642	0.0018907	0.0732949	-3.669512	ALS
CTSL	-612.9311	1708.3483	-3.600483	0.0019521	0.0732949	-3.674724	ALS
CD209	-64.0523	66.82915	-3.582092	0.0020349	0.0732949	-3.681509	ALS
PPARG	-72.5951	140.50212	-3.576394	0.0020612	0.0732949	-3.683615	ALS
TNFRSF1A	-580.4968	1530.0789	-3.574662	0.0020693	0.0732949	-3.684255	ALS
IL15	-46.65435	84.842975	-3.559771	0.00214	0.0732949	-3.689767	ALS
OSCAR	-129.0814	132.0092	-3.481872	0.0025507	0.083868	-3.718781	ALS
TLR2	-33.0427	47.29645	-3.429941	0.0028669	0.0875055	-3.738285	ALS

SLC11A1	-674.7893	914.0818	-3.428793	0.0028743	0.0875055	-3.738718	ALS
CASP4	-67.9586	146.22637	-3.374119	0.0032498	0.0954048	-3.759388	ALS
AIRE	-66.25085	55.619825	-3.344431	0.0034735	0.0984566	-3.770666	ALS
LY9	-12.63885	35.004775	-3.322497	0.0036485	0.0989358	-3.779023	ALS
SPPL2A	-166.5511	605.16275	-3.312489	0.0037312	0.0989358	-3.782842	ALS
SH2D1A	-37.3321	106.81895	-3.287294	0.0039475	0.1014019	-3.792476	ALS
CCR2	-10.42075	18.770475	-3.251322	0.0042779	0.1065584	-3.806274	ALS
MX1	-1418.249	4278.2718	-3.226008	0.0045265	0.109434	-3.816013	ALS
CD300LF	-94.1562	166.625	-3.15654	0.0052834	0.1158614	-3.842857	ALS
C5AR1	-178.8921	255.32725	-3.139053	0.0054927	0.1158614	-3.84964	ALS
POLR3F	75.4675	355.14075	3.1013602	0.0059715	0.1158614	-3.864293	ALS
TREM1	-32.6688	45.2648	-3.093384	0.0060779	0.1158614	-3.8674	ALS
CTSS	-184.1474	266.7035	-3.090861	0.0061119	0.1158614	-3.868383	ALS
NCF2	-118.0496	139.6378	-3.085057	0.006191	0.1158614	-3.870645	ALS
CACNA1C	-87.0408	230.7261	-3.074262	0.0063406	0.1158614	-3.874855	ALS
IFNG	-13.8752	34.089	-3.06854	0.0064213	0.1158614	-3.877088	ALS
CCL24	10.2603	11.38405	3.0553069	0.0066118	0.1158614	-3.882255	ALS
HIST2H2BE	377.2131	1044.3544	3.0505512	0.0066816	0.1158614	-3.884113	ALS
PRKD1	183.4644	546.4851	3.0472985	0.0067298	0.1158614	-3.885384	ALS
HIST1H2BK	604.9025	2186.444	3.0409319	0.006825	0.1158614	-3.887873	ALS
TNFRSF10B	-118.4651	361.39125	-3.034467	0.0069231	0.1158614	-3.890402	ALS
HLA-B	-1073.516	2963.1202	-3.024572	0.0070758	0.1158614	-3.894274	ALS
RELB	-25.1322	54.1259	-3.024085	0.0070834	0.1158614	-3.894465	ALS
FCER1G	-1293.353	2355.4195	-3.021816	0.0071189	0.1158614	-3.895353	ALS
DEFA3	90.9919	48.46885	3.0018248	0.0074394	0.1158614	-3.903185	ALS
BTN3A1	-31.8923	155.968	-3.001645	0.0074423	0.1158614	-3.903256	ALS
ELF4	-88.7936	229.4115	-2.970486	0.00797	0.1158614	-3.915482	ALS
GBP2	-1368.903	2333.4878	-2.960001	0.0081556	0.1158614	-3.919601	ALS
FCGR2B	-198.2464	181.6314	-2.952367	0.0082934	0.1158614	-3.922602	ALS
IL4R	-1016.239	2131.3638	-2.943561	0.0084551	0.1158614	-3.926064	ALS
CHIT1	-145.2391	131.18905	-2.940471	0.0085125	0.1158614	-3.92728	ALS
ADAM15	-1104.213	5107.7815	-2.935273	0.00861	0.1158614	-3.929325	ALS

GBF1	-31.7614	243.7149	-2.931672	0.0086782	0.1158614	-3.930742	ALS
IRAK1	-295.2539	1350.4378	-2.931622	0.0086791	0.1158614	-3.930762	ALS
HLA-A	-894.9333	3117.8166	-2.929807	0.0087137	0.1158614	-3.931476	ALS
IL18RAP	-85.7225	158.46325	-2.922977	0.008845	0.1158614	-3.934165	ALS
ETS1	-367.0895	1143.4596	-2.921176	0.0088799	0.1158614	-3.934874	ALS
SAMSN1	-188.122	340.9688	-2.906835	0.0091628	0.1161511	-3.940522	ALS
AQP9	-244.9315	213.51655	-2.905743	0.0091847	0.1161511	-3.940952	ALS
KLRG1	33.5596	168.6617	2.8862458	0.0095842	0.1193674	-3.948638	ALS
HLA-F	-111.2457	301.47955	-2.83214	0.0107826	0.1318157	-3.969997	ALS
GBP5	-14.7759	27.84045	-2.823256	0.0109926	0.1318157	-3.973508	ALS
TOLLIP	83.6271	506.29325	2.8202394	0.0110648	0.1318157	-3.9747	ALS
LY96	-906.4126	1626.5215	-2.810715	0.0112958	0.1321624	-3.978466	ALS
IL7	-10.8774	17.5502	-2.805854	0.0114155	0.1321624	-3.980388	ALS
DEFA1	125.4485	88.43565	2.791972	0.011764	0.1343052	-3.985878	ALS
FES	-270.1117	500.87745	-2.747775	0.0129426	0.1438443	-4.003369	ALS
IL1RL2	-9.8884	20.7842	-2.747528	0.0129495	0.1438443	-4.003467	ALS
SRPK2	53.6397	257.56265	2.7161746	0.0138537	0.1518363	-4.015883	ALS
GEM	16.3448	50.1583	2.6966923	0.0144455	0.1562397	-4.023599	ALS
CD84	-109.1958	157.9283	-2.684815	0.0148181	0.1566526	-4.028304	ALS
PLSCR1	-214.1514	346.5814	-2.670374	0.0152834	0.1566526	-4.034025	ALS
CCR7	-23.2563	20.12345	-2.667748	0.0153695	0.1566526	-4.035065	ALS
DAB2IP	-12.77655	61.333275	-2.663962	0.0154944	0.1566526	-4.036565	ALS
IGF1R	19.1435	53.94795	2.6546113	0.0158071	0.1566526	-4.040269	ALS
KCNN4	-86.0446	102.9747	-2.65033	0.0159523	0.1566526	-4.041965	ALS
ICAM3	-116.0064	444.5772	-2.649402	0.0159839	0.1566526	-4.042332	ALS
AZGP1	153.8667	200.64775	2.6486892	0.0160083	0.1566526	-4.042615	ALS
NFKB2	-38.0884	117.8193	-2.635731	0.0164569	0.1591482	-4.047747	ALS
CEBPG	-241.397	1318.5822	-2.589505	0.0181565	0.1716715	-4.066052	ALS
IFI16	-441.1212	981.1454	-2.589166	0.0181696	0.1716715	-4.066186	ALS
ZBP1	-34.6595	94.69515	-2.56828	0.0189914	0.1773971	-4.074452	ALS
POLR3K	47.931	265.6699	2.5380187	0.0202447	0.1869789	-4.086421	ALS
TRIM31	10.4888	16.9629	2.5133648	0.0213229	0.1926954	-4.096165	ALS

CMKLR1	-43.4618	83.1903	-2.508309	0.0215506	0.1926954	-4.098162	ALS
APOBEC3B	7.9904	17.0911	2.50795	0.0215669	0.1926954	-4.098304	ALS
AIM2	-42.4674	135.0682	-2.482367	0.0227554	0.196184	-4.108403	ALS
COLEC12	-180.9078	476.3019	-2.47595	0.023063	0.196184	-4.110934	ALS
LRMP	-138.6325	318.59925	-2.473758	0.023169	0.196184	-4.111799	ALS
SECTM1	-10.3745	31.72965	-2.468862	0.0234073	0.196184	-4.11373	ALS
CEACAM8	14.7464	14.8841	2.4675195	0.0234731	0.196184	-4.114259	ALS
TYROBP	-1544.57	3484.8247	-2.459478	0.0238706	0.196184	-4.11743	ALS
SLPI	146.7319	119.83775	2.4577018	0.0239593	0.196184	-4.11813	ALS
HLA-DOA	-284.5127	502.68555	-2.45539	0.0240751	0.196184	-4.119041	ALS
KLRF1	-11.1908	23.0779	-2.454789	0.0241053	0.196184	-4.119278	ALS
TLR8	-35.89805	68.335275	-2.445182	0.024593	0.1981903	-4.123062	ALS
TNFRSF14	-945.9218	2319.8343	-2.392082	0.0274589	0.2167127	-4.143945	ALS
BTK	-453.5092	803.8258	-2.390155	0.0275686	0.2167127	-4.144702	ALS
FYB	-284.2574	537.22138	-2.388165	0.0276823	0.2167127	-4.145484	ALS
HLA-DMB	-2306.818	5173.5692	-2.377812	0.0282807	0.2180811	-4.149546	ALS
LYN	-607.8534	1330.0651	-2.375982	0.0283877	0.2180811	-4.150264	ALS
TAP1	-609.7211	2061.5686	-2.364997	0.0290381	0.2193469	-4.154571	ALS
HAVCR2	-1866.832	4633.1184	-2.352831	0.0297745	0.2193469	-4.159337	ALS
AXL	-229.6498	1083.1842	-2.351165	0.0298767	0.2193469	-4.159989	ALS
LIF	-21.9937	50.11725	-2.351037	0.0298846	0.2193469	-4.160039	ALS
DEFA5	10.8891	20.95915	2.3510027	0.0298867	0.2193469	-4.160052	ALS
DEFB1	14.1226	18.9795	2.3375059	0.0307269	0.2235179	-4.165334	ALS
PIK3CG	-108.3183	215.74665	-2.322247	0.0317032	0.2280597	-4.171297	ALS
PTPN22	-12.2577	28.90135	-2.31913	0.0319062	0.2280597	-4.172515	ALS
CTSG	17.3788	29.8632	2.3119465	0.0323785	0.2291241	-4.175319	ALS
CTSC	-97.8116	275.89755	-2.308422	0.0326126	0.2291241	-4.176694	ALS
GZMA	-75.4505	167.01555	-2.294012	0.033586	0.2339633	-4.182313	ALS
CD1D	-76.1471	151.33515	-2.287708	0.0340202	0.234997	-4.184768	ALS
LCP1	-705.3193	2176.9277	-2.249061	0.0367977	0.252014	-4.199789	ALS
BCL2	76.22865	341.77198	2.2450596	0.0370969	0.252014	-4.201341	ALS
CR2	-5.4298	12.0121	-2.230476	0.0382065	0.2543018	-4.206991	ALS

CXCL2	186.8747	143.67565	2.2291089	0.0383121	0.2543018	-4.207521	ALS
TNFSF14	-14.964	30.3743	-2.221609	0.0388959	0.2543018	-4.210423	ALS
CD7	-37.377	112.5229	-2.221097	0.0389361	0.2543018	-4.210621	ALS
INPP5D	-326.2064	1003.2787	-2.217175	0.039245	0.2543018	-4.212137	ALS
GPR65	-86.6909	117.04365	-2.215996	0.0393383	0.2543018	-4.212593	ALS
IL19	4.70135	17.207025	2.2117212	0.0396782	0.2543018	-4.214245	ALS
CLEC4A	-31.2721	74.21565	-2.208842	0.0399087	0.2543018	-4.215357	ALS
FCER2	-7.8041	34.75345	-2.197471	0.040831	0.2558232	-4.219745	ALS
KLRB1	-13.1887	35.91235	-2.195478	0.0409947	0.2558232	-4.220514	ALS
SELL	93.3124	149.5029	2.1944288	0.0410811	0.2558232	-4.220918	ALS
RIPK3	-31.1561	65.53305	-2.182183	0.0421019	0.2602088	-4.225636	ALS
POLR3H	84.5669	403.5745	2.1762605	0.0426038	0.2610907	-4.227915	ALS
ADAR	-284.4994	2136.572	-2.170831	0.0430688	0.2610907	-4.230003	ALS
ATG12	-58.3273	290.81485	-2.168835	0.0432409	0.2610907	-4.23077	ALS
ENPP2	1296.5725	4875.332	2.1656699	0.0435151	0.2610907	-4.231986	ALS
POLR3E	-35.773	338.7366	-2.158954	0.0441022	0.2622049	-4.234565	ALS
TRPM4	-95.6391	462.36905	-2.149734	0.0449199	0.2622049	-4.238102	ALS
CD33	-104.3681	260.40675	-2.148466	0.0450334	0.2622049	-4.238588	ALS
ITGB7	14.6824	39.8181	2.1468476	0.0451787	0.2622049	-4.239208	ALS
TAPBP	-66.90963	410.31385	-2.141076	0.0457003	0.2622049	-4.241418	ALS
KLRC2	-6.4527	18.24145	-2.137321	0.0460426	0.2622049	-4.242856	ALS
GZMH	-74.9795	139.51405	-2.135433	0.0462157	0.2622049	-4.243579	ALS
CAPZA2	-414.0682	3960.8522	-2.13503	0.0462527	0.2622049	-4.243733	ALS
DOCK2	-570.9506	1380.1867	-2.122891	0.0473799	0.2640129	-4.248372	ALS
ZP4	10.5593	29.05955	2.1223706	0.0474288	0.2640129	-4.248571	ALS
WAS	-765.3475	2051.9168	-2.12124	0.0475352	0.2640129	-4.249003	ALS
CD8A	-56.2226	106.4285	-2.115907	0.0480397	0.2650246	-4.251038	ALS
LAIR2	-39.3587	70.92715	-2.098665	0.0497046	0.2723814	-4.257609	ALS
RNF135	-60.6579	94.28425	-6.412027	4.11E-06	0.0020049	-2.565256	HD
COCH	5018.3475	4222.3392	6.1780926	6.66E-06	0.0020049	-2.623174	HD
STYK1	68.2473	58.05895	6.1326518	7.32E-06	0.0020049	-2.634819	HD
EOMES	-32.2597	38.07335	-5.683036	1.89E-05	0.0036713	-2.757304	HD

**CAMK4**	**221.2182**	**191.7856**	**5.6050757**	**2.23E-05**	**0.0036713**	**-2.779934**	**HD**
**FOXJ1**	**-1213.061**	**838.37985**	**-5.327509**	**4.07E-05**	**0.0054141**	**-2.864004**	**HD**
**PLCL2**	**817.7155**	**1198.5585**	**5.2240766**	**5.10E-05**	**0.0054141**	**-2.896759**	**HD**
**F12**	**680.2108**	**722.6024**	**5.1716585**	**5.72E-05**	**0.0054141**	**-2.91366**	**HD**
**DRD2**	**170.9378**	**205.252**	**5.0759348**	**7.06E-05**	**0.0054141**	**-2.945052**	**HD**
**EIF2AK2**	**-959.6979**	**2885.7454**	**-5.024209**	**7.92E-05**	**0.0054141**	**-2.962301**	**HD**
**POLR3H**	**-366.593**	**504.89543**	**-5.020711**	**7.98E-05**	**0.0054141**	**-2.963475**	**HD**
**FKBP1A**	**1626.5405**	**2100.3226**	**5.0072553**	**8.22E-05**	**0.0054141**	**-2.967998**	**HD**
**TNFSF4**	**-31.0119**	**53.21165**	**-4.988793**	**8.56E-05**	**0.0054141**	**-2.974227**	**HD**
**GPI**	**1096.7866**	**3193.5496**	**4.8021185**	**0.0001296**	**0.007207**	**-3.038657**	**HD**
**NOTCH1**	**-964.2755**	**1580.7742**	**-4.795518**	**0.0001315**	**0.007207**	**-3.040984**	**HD**
**CCR6**	**17.4193**	**26.86605**	**4.7349604**	**0.0001505**	**0.0077338**	**-3.062485**	**HD**
**FCGRT**	**-434.0735**	**724.14745**	**-4.648522**	**0.0001826**	**0.0088311**	**-3.093659**	**HD**
**CX3CL1**	**1890.3498**	**2664.4083**	**4.6004985**	**0.0002034**	**0.0092882**	**-3.111225**	**HD**
**CD8A**	**71.5358**	**110.2161**	**4.5082229**	**0.0002502**	**0.0099241**	**-3.14547**	**HD**
**HERC5**	**-832.686**	**1199.1748**	**-4.495659**	**0.0002574**	**0.0099241**	**-3.150183**	**HD**
**ANGPT1**	**-359.677**	**267.2579**	**-4.494426**	**0.0002581**	**0.0099241**	**-3.150646**	**HD**
**ADARB1**	**235.8865**	**600.8322**	**4.4783604**	**0.0002676**	**0.0099241**	**-3.156691**	**HD**
**SLC25A6**	**926.2757**	**2471.6735**	**4.4518367**	**0.0002841**	**0.0099241**	**-3.166714**	**HD**
**IKBKB**	**-195.4322**	**410.4516**	**-4.429294**	**0.0002989**	**0.0099241**	**-3.175275**	**HD**
**BCL2**	**-262.361**	**433.2608**	**-4.424909**	**0.0003018**	**0.0099241**	**-3.176944**	**HD**
**ZC3HAV1**	**-322.4778**	**512.48075**	**-4.329075**	**0.0003746**	**0.0116893**	**-3.213795**	**HD**
**PRKCD**	**248.0837**	**434.62395**	**4.3182058**	**0.000384**	**0.0116893**	**-3.218018**	**HD**
**PRKD2**	**-398.3132**	**659.1114**	**-4.279278**	**0.0004192**	**0.0119596**	**-3.233215**	**HD**
**CTSH**	**-165.9757**	**204.66255**	**-4.276451**	**0.0004219**	**0.0119596**	**-3.234323**	**HD**
**FYN**	**-140.0463**	**471.60203**	**-4.235314**	**0.000463**	**0.0126874**	**-3.250512**	**HD**
**ITGB7**	**-14.1646**	**24.5815**	**-4.219106**	**0.0004803**	**0.0127365**	**-3.256925**	**HD**
**PPP1R14B**	**-1397.593**	**2288.5461**	**-4.185569**	**0.0005182**	**0.013311**	**-3.270256**	**HD**
**ABL1**	**-176.0144**	**382.2134**	**-4.164968**	**0.0005429**	**0.0135236**	**-3.278486**	**HD**
**ICAM5**	**264.694**	**296.6232**	**4.0231244**	**0.0007486**	**0.0180982**	**-3.335979**	**HD**
**BCL6**	**-1250.215**	**1771.5457**	**-4.002537**	**0.0007843**	**0.0184207**	**-3.344443**	**HD**
**OPRK1**	**689.543**	**637.3947**	**3.9784535**	**0.0008283**	**0.0184589**	**-3.354381**	**HD**

TNFRSF1A	-606.3877	814.19775	-3.977099	0.0008309	0.0184589	-3.354941	HD
TNFRSF8	34.0594	46.8106	3.9466433	0.0008902	0.0192575	-3.36757	HD
NFKB2	-62.3747	95.20105	-3.818643	0.0011898	0.0250778	-3.421334	HD
COL17A1	125.5419	224.03475	3.8068671	0.001222	0.0251119	-3.426334	HD
CSK	-451.6573	684.00975	-3.758383	0.0013638	0.027343	-3.447019	HD
CD81	-7946.581	26929.678	-3.734816	0.0014386	0.0275307	-3.457128	HD
SUSD4	1236.9187	1761.1333	3.7343243	0.0014402	0.0275307	-3.457339	HD
TNFRSF11B	-135.258	118.3098	-3.703567	0.001544	0.0288447	-3.470585	HD
AXL	-473.5737	1272.9068	-3.687599	0.0016008	0.0291894	-3.477485	HD
SERPING1	-157.2416	276.0657	-3.67867	0.0016335	0.0291894	-3.48135	HD
TNFRSF10B	-97.1702	166.4734	-3.601604	0.0019444	0.0340055	-3.514911	HD
ALOX15	10.2847	12.61395	3.5675763	0.0020997	0.0359567	-3.52984	HD
YES1	-617.263	957.4977	-3.537934	0.0022449	0.0376596	-3.542899	HD
SOX9	-1182.491	2231.5574	-3.521954	0.0023273	0.0382609	-3.54996	HD
CD200	116.3319	206.98165	3.4899705	0.0025013	0.0403148	-3.564133	HD
NUDCD1	65.5794	111.6406	3.4517426	0.0027261	0.0425035	-3.581146	HD
POLR3C	264.0639	766.63835	3.4494097	0.0027405	0.0425035	-3.582186	HD
TLR4	-92.5067	169.77315	-3.415868	0.0029552	0.0449854	-3.59718	HD
CXADR	481.1201	1124.095	3.4072498	0.0030131	0.0450314	-3.601041	HD
LGR4	-582.829	956.4656	-3.387445	0.0031501	0.0462396	-3.609929	HD
ABL2	13.77605	39.100225	3.3536319	0.0033985	0.0490102	-3.625148	HD
TRIM11	122.3136	392.2641	3.3391852	0.0035104	0.0492179	-3.631666	HD
ITGB1	-450.8117	1149.8173	-3.335829	0.0035369	0.0492179	-3.633182	HD
F2RL1	-59.9845	76.72195	-3.328868	0.0035925	0.0492179	-3.636328	HD
TRIM8	-754.1941	3849.9063	-3.295892	0.0038679	0.0520394	-3.651258	HD
TGFBR3	-1160.306	1284.9656	-3.289328	0.0039251	0.0520394	-3.654236	HD
POLR3B	145.0566	687.2547	3.2377477	0.0044046	0.0570972	-3.6777	HD
RFX1	-209.2025	475.19875	-3.233608	0.0044455	0.0570972	-3.679588	HD
ANG	-261.1589	375.42965	-3.226235	0.0045193	0.0571513	-3.682952	HD
OTUB1	17.5505	65.76765	3.2003599	0.0047876	0.0596277	-3.694775	HD
TICAM2	-175.4692	267.8852	-3.164903	0.0051808	0.0635614	-3.711018	HD
ANXA1	-1779.673	1603.8263	-3.123431	0.0056807	0.0678137	-3.730074	HD

**TICAM1**	**103.4452**	**425.50625**	**3.1184925**	**0.0057433**	**0.0678137**	**-3.732347**	**HD**
**INPPL1**	**-840.3174**	**1267.79**	**-3.114757**	**0.0057911**	**0.0678137**	**-3.734067**	**HD**
**POLR3A**	**43.2989**	**248.19695**	**3.1096235**	**0.0058574**	**0.0678137**	**-3.736432**	**HD**
**INPP5D**	**-265.0877**	**413.13505**	**-3.0915**	**0.0060974**	**0.0696119**	**-3.744786**	**HD**
**POLR3K**	**147.7018**	**334.7388**	**3.0632654**	**0.0064905**	**0.0730845**	**-3.757821**	**HD**
**TNFSF12**	**-120.798**	**452.46965**	**-3.02253**	**0.0071013**	**0.0778468**	**-3.776667**	**HD**
**NFKB1**	**-573.1618**	**1747.17**	**-3.017745**	**0.0071766**	**0.0778468**	**-3.778884**	**HD**
**CHGA**	**528.1973**	**834.51915**	**3.0164236**	**0.0071975**	**0.0778468**	**-3.779496**	**HD**
**CRIP1**	**-1260.183**	**1424.2266**	**-2.978138**	**0.0078304**	**0.0835917**	**-3.797256**	**HD**
**HRAS**	**153.125**	**555.3265**	**2.954854**	**0.0082412**	**0.0868498**	**-3.808072**	**HD**
**PTK2B**	**105.04895**	**182.02343**	**2.9417785**	**0.008481**	**0.0871041**	**-3.814152**	**HD**
**IFNW1**	**-8.0544**	**20.1428**	**-2.940828**	**0.0084987**	**0.0871041**	**-3.814594**	**HD**
**IL27RA**	**-39.4837**	**103.23935**	**-2.934233**	**0.0086224**	**0.0871041**	**-3.817662**	**HD**
**TRPM4**	**-81.8035**	**251.64025**	**-2.930711**	**0.0086892**	**0.0871041**	**-3.819301**	**HD**
**SRPK2**	**164.59285**	**447.22383**	**2.9072476**	**0.0091471**	**0.0905892**	**-3.830224**	**HD**
**TNFRSF10D**	**-32.8936**	**40.9237**	**-2.890126**	**0.0094958**	**0.0929232**	**-3.838201**	**HD**
**S100B**	**-392.6748**	**598.0017**	**-2.880813**	**0.0096908**	**0.0936752**	**-3.842541**	**HD**
**CD96**	**-7.7859**	**15.54675**	**-2.875651**	**0.0098006**	**0.0936752**	**-3.844948**	**HD**
**IL13RA2**	**154.6031**	**242.26195**	**2.8658955**	**0.0100112**	**0.094589**	**-3.849497**	**HD**
**EXOSC9**	**115.9333**	**383.59945**	**2.8212029**	**0.0110334**	**0.1024186**	**-3.870355**	**HD**
**FTH1**	**1213.9432**	**4331.514**	**2.8188833**	**0.0110891**	**0.1024186**	**-3.871438**	**HD**
**TNFRSF14**	**-565.3504**	**1044.5852**	**-2.806894**	**0.0113814**	**0.1039498**	**-3.877037**	**HD**
**TGFB2**	**-38.2109**	**62.12335**	**-2.781166**	**0.0120336**	**0.108699**	**-3.889057**	**HD**
**PCBP2**	**-1039.911**	**3739.3827**	**-2.770176**	**0.012323**	**0.1101032**	**-3.894192**	**HD**
**HSPD1**	**-569.1446**	**1001.8793**	**-2.759341**	**0.0126148**	**0.1114985**	**-3.899256**	**HD**
**PTX3**	**13.633**	**54.4379**	**2.7145791**	**0.0138919**	**0.1191382**	**-3.920181**	**HD**
**TRIM6**	**-16.5235**	**21.83555**	**-2.711027**	**0.0139984**	**0.1191382**	**-3.921842**	**HD**
**RPS6**	**-2686.024**	**14800.345**	**-2.709923**	**0.0140316**	**0.1191382**	**-3.922358**	**HD**
**CXCL12**	**-108.2519**	**186.76658**	**-2.70902**	**0.0140589**	**0.1191382**	**-3.92278**	**HD**
**CLEC2B**	**-103.4116**	**129.1311**	**-2.692605**	**0.0145631**	**0.1200601**	**-3.930454**	**HD**
**CD36**	**-48.35085**	**61.773075**	**-2.6875**	**0.0147234**	**0.1200601**	**-3.932841**	**HD**
**CD3E**	**-8.2634**	**16.4069**	**-2.687022**	**0.0147385**	**0.1200601**	**-3.933064**	**HD**

JAK2	36.0414	173.2404	2.6865987	0.0147519	0.1200601	-3.933262	HD
IL2	11.4665	33.76235	2.6665796	0.0153981	0.1209579	-3.94262	HD
IKBKAP	23.3983	71.32425	2.6663737	0.0154049	0.1209579	-3.942716	HD
TFE3	-80.8345	273.09645	-2.663543	0.0154984	0.1209579	-3.94404	HD
CLU	-37.0824	123.13245	-2.657433	0.0157022	0.1209579	-3.946896	HD
ITK	26.6386	32.9192	2.6534752	0.0158355	0.1209579	-3.948745	HD
BMPR1A	-216.9818	408.8712	-2.644105	0.0161556	0.1209579	-3.953124	HD
TCF12	-159.3126	483.6879	-2.643809	0.0161658	0.1209579	-3.953263	HD
TYK2	-548.6246	1882.0184	-2.641429	0.0162481	0.1209579	-3.954375	HD
ENPP3	-16.9517	43.42375	-2.630738	0.0166227	0.1209579	-3.95937	HD
HMGB3	-53.1842	177.3247	-2.625691	0.0168025	0.1209579	-3.961728	HD
CD209	13.2394	17.2743	2.625552	0.0168074	0.1209579	-3.961793	HD
COLEC12	-452.3561	618.63623	-2.624908	0.0168305	0.1209579	-3.962094	HD
CTSW	-11.3448	17.248	-2.623967	0.0168642	0.1209579	-3.962533	HD
CD6	-10.1717	28.36905	-2.622352	0.0169223	0.1209579	-3.963288	HD
ALCAM	461.7982	2681.7578	2.6058414	0.0175272	0.1242016	-3.970999	HD
BIRC2	-29.007	192.9667	-2.572022	0.0188308	0.1322984	-3.986786	HD
PRKD1	-299.0586	667.5201	-2.564411	0.0191365	0.133307	-3.990337	HD
MARCO	-14.7918	35.1032	-2.549497	0.0197493	0.1364192	-3.997292	HD
C3	-1303.051	1386.8306	-2.535999	0.0203198	0.1366753	-4.003585	HD
SAA1	-8.68925	14.969875	-2.535383	0.0203462	0.1366753	-4.003871	HD
TRAF3IP2	-89.8527	378.4587	-2.534495	0.0203843	0.1366753	-4.004285	HD
HLA-DQA2	-28.8472	60.5975	-2.532935	0.0204514	0.1366753	-4.005012	HD
CSF1	-8.4914	22.9593	-2.525924	0.0207557	0.1375899	-4.008279	HD
ETS1	-341.1578	807.5262	-2.518507	0.0210822	0.1378194	-4.011733	HD
APOBEC3F	-13.7581	42.73875	-2.517529	0.0211256	0.1378194	-4.012189	HD
AZGP1	-88.7246	118.0128	-2.512988	0.0213283	0.138046	-4.014303	HD
TLR5	-259.3343	341.79725	-2.487139	0.0225173	0.1430863	-4.026331	HD
PIK3CG	-49.9254	61.513	-2.486016	0.0225704	0.1430863	-4.026853	HD
VIPR1	190.1666	393.9928	2.4847726	0.0226292	0.1430863	-4.027432	HD
CFI	-152.0165	325.14765	-2.480086	0.0228524	0.1433945	-4.029611	HD
IRAK1	-279.4803	823.16215	-2.450431	0.0243132	0.1507329	-4.043385	HD

**BST2**	**-513.0496**	**961.0911**	**-2.448945**	**0.0243887**	**0.1507329**	**-4.044075**	**HD**
**ZBP1**	**-13.3124**	**45.5113**	**-2.424253**	**0.0256749**	**0.1567515**	**-4.055526**	**HD**
**DCLRE1C**	**-71.7769**	**196.10605**	**-2.422963**	**0.0257439**	**0.1567515**	**-4.056123**	**HD**
**CEBPG**	**-820.8982**	**1675.5593**	**-2.409302**	**0.0264843**	**0.1600744**	**-4.06245**	**HD**
**CYBA**	**-277.2948**	**765.2584**	**-2.399099**	**0.0270503**	**0.1623019**	**-4.067172**	**HD**
**AMBP**	**-122.6215**	**117.79145**	**-2.384093**	**0.0279032**	**0.1662063**	**-4.07411**	**HD**
**PIK3CD**	**-71.1037**	**136.29955**	**-2.372009**	**0.0286082**	**0.1691797**	**-4.079691**	**HD**
**TNFRSF21**	**474.6264**	**1658.6276**	**2.3610929**	**0.0292593**	**0.1706665**	**-4.084728**	**HD**
**PGLYRP4**	**-15.3793**	**41.67855**	**-2.36001**	**0.0293246**	**0.1706665**	**-4.085228**	**HD**
**IFIH1**	**-178.7926**	**360.3068**	**-2.357402**	**0.0294825**	**0.1706665**	**-4.08643**	**HD**
**TRIM26**	**-241.0014**	**557.8489**	**-2.346912**	**0.0301257**	**0.1731699**	**-4.091265**	**HD**
**HLA-F**	**78.872**	**202.6006**	**2.3408935**	**0.0305005**	**0.1732457**	**-4.094037**	**HD**
**TFEB**	**-24.551**	**49.637**	**-2.339939**	**0.0305604**	**0.1732457**	**-4.094477**	**HD**
**TRIM32**	**48.4568**	**194.6869**	**2.332636**	**0.0310219**	**0.1732896**	**-4.097838**	**HD**
**TNFSF9**	**-24.1484**	**84.1585**	**-2.331649**	**0.0310848**	**0.1732896**	**-4.098292**	**HD**
**CLEC5A**	**-61.2764**	**80.4433**	**-2.329836**	**0.0312006**	**0.1732896**	**-4.099127**	**HD**
**SIRT1**	**-203.4723**	**667.22365**	**-2.32569**	**0.0314668**	**0.1735954**	**-4.101033**	**HD**
**VTCN1**	**8.1834**	**12.352**	**2.3179612**	**0.0319689**	**0.1742255**	**-4.104586**	**HD**
**C4B**	**-55.7027**	**47.82565**	**-2.31721**	**0.0320181**	**0.1742255**	**-4.104932**	**HD**
**TRIM35**	**18.221**	**57.3663**	**2.3141845**	**0.0322169**	**0.1742255**	**-4.106321**	**HD**
**LAT2**	**-36.5965**	**91.01865**	**-2.310318**	**0.0324726**	**0.1744607**	**-4.108097**	**HD**
**C4BPA**	**-10.8058**	**35.7885**	**-2.306145**	**0.0327508**	**0.1748125**	**-4.110013**	**HD**
**CACNA1C**	**82.4642**	**283.6249**	**2.3004641**	**0.0331329**	**0.1757115**	**-4.11262**	**HD**
**ELF4**	**-44.1753**	**99.08845**	**-2.287478**	**0.0340221**	**0.1792703**	**-4.118574**	**HD**
**AQP3**	**6.8471**	**15.47625**	**2.2203941**	**0.0389765**	**0.2040682**	**-4.1492**	**HD**
**C6**	**-73.1272**	**67.5621**	**-2.213258**	**0.0395408**	**0.2057124**	**-4.152444**	**HD**
**SIRT2**	**-115.4012**	**393.646**	**-2.200812**	**0.0405431**	**0.2096001**	**-4.158095**	**HD**
**HIST1H2BC**	**-7.826**	**30.9378**	**-2.183346**	**0.0419888**	**0.2140943**	**-4.16601**	**HD**
**GPR65**	**-21.8301**	**32.34755**	**-2.177369**	**0.0424942**	**0.2140943**	**-4.168715**	**HD**
**ZNF683**	**-17.4875**	**88.60225**	**-2.177053**	**0.0425211**	**0.2140943**	**-4.168858**	**HD**
**MARCH1**	**23.1256**	**77.4263**	**2.1760756**	**0.0426044**	**0.2140943**	**-4.1693**	**HD**
**SERINC5**	**12.2461**	**38.00535**	**2.1747829**	**0.0427147**	**0.2140943**	**-4.169884**	**HD**

GEM	-114.168	138.1884	-2.169909	0.043133	0.2148807	-4.172088	HD
AQP9	83.1664	150.1352	2.1623562	0.0437885	0.2168323	-4.175499	HD
CRISP3	8.0878	29.4821	2.1518562	0.044715	0.2200942	-4.180235	HD
PML	-18.96783	69.936583	-2.145295	0.045303	0.2210674	-4.183191	HD
IRF7	-74.5301	185.29625	-2.14366	0.0454506	0.2210674	-4.183927	HD
IL32	-32.2542	41.2417	-2.127618	0.0469223	0.2268832	-4.191141	HD
IL18	-8571.919	27074.689	-2.122048	0.0474433	0.2280609	-4.193641	HD
ZC3HAV1	-88.1817	224.1674	-5.216923	5.09E-05	0.0418681	-2.27266	MS
IKBKG	184.3581	886.19235	4.2923161	0.0004026	0.1279114	-2.739319	MS
SEMA4D	283.9253	1124.201	4.2269982	0.0004668	0.1279114	-2.775461	MS
RPS6	-1295.2	9468.3933	-3.99423	0.0007919	0.1524266	-2.907464	MS
BCL6	-710.8964	1482.2892	-3.924798	0.0009272	0.1524266	-2.947773	MS
PRG4	14.7018	13.1447	3.7686994	0.0013217	0.1810739	-3.039869	MS
PRKCQ	86.4608	226.3537	3.5661496	0.0020919	0.2456451	-3.162186	MS
PRG2	73.67745	249.28663	3.450852	0.0027143	0.2788933	-3.23308	MS
TNFRSF1A	-261.5153	579.31385	-3.277054	0.0040121	0.3294108	-3.341424	MS
ADAR	-240.0305	2340.6687	-3.163039	0.0051762	0.3294108	-3.413301	MS
KIR2DS5	-10.3922	27.0968	-3.156963	0.0052467	0.3294108	-3.417146	MS
APLN	-35.7575	84.72035	-3.120242	0.0056934	0.3294108	-3.440414	MS
POLR3C	212.9002	708.2757	3.1159166	0.0057484	0.3294108	-3.443158	MS
CYBA	-222.2897	400.64415	-3.081947	0.0061986	0.3294108	-3.464727	MS
HLA-DQA2	-14.0768	49.7993	-3.079579	0.0062312	0.3294108	-3.466232	MS
TICAM2	-64.3868	202.2965	-3.051014	0.0066383	0.3294108	-3.484399	MS
CD83	165.9426	778.55	3.0392937	0.0068126	0.3294108	-3.491859	MS
LY96	-170.0409	336.69985	-2.937792	0.0085204	0.3511067	-3.5566	MS
IFITM3	-2382.268	3846.1494	-2.927653	0.0087121	0.3511067	-3.563077	MS
TGFB2	-17.477	42.6896	-2.922487	0.0088114	0.3511067	-3.566378	MS
CD81	-2630.733	15295.87	-2.899783	0.0092608	0.3511067	-3.580889	MS
CTSS	-23.4522	39.6869	-2.817086	0.0110919	0.3511067	-3.633782	MS
CX3CL1	612.031	3048.0654	2.7998163	0.0115159	0.3511067	-3.644831	MS
IFITM2	-3236.718	5313.853	-2.798733	0.011543	0.3511067	-3.645524	MS
HIST2H2BE	-169.2914	420.8823	-2.796633	0.0115957	0.3511067	-3.646867	MS

TNFAIP1	110.2817	943.21685	2.7965486	0.0115979	0.3511067	-3.646921	MS
RPL39	-940.2404	6678.3546	-2.792947	0.0116889	0.3511067	-3.649226	MS
MICB	-58.0567	77.86685	-2.770459	0.0122726	0.3511067	-3.663613	MS
ENPP2	470.0336	852.7743	2.7209183	0.0136585	0.3511067	-3.695297	MS
CD226	-10.4389	20.80655	-2.68383	0.0147925	0.3511067	-3.719001	MS
ANXA1	-163.7771	435.23005	-2.682582	0.0148322	0.3511067	-3.719799	MS
SAA1	-7.54035	14.173625	-2.644745	0.0160843	0.3511067	-3.743958	MS
CD24	14.327	33.1892	2.6383189	0.0163067	0.3511067	-3.748059	MS
LILRB5	-12.3247	31.42985	-2.632341	0.0165161	0.3511067	-3.751873	MS
TRIM5	-16.33015	33.872975	-2.626561	0.0167211	0.3511067	-3.755559	MS
ABL1	-21.5288	196.7206	-2.619403	0.0169783	0.3511067	-3.760124	MS
SFTPD	85.5112	256.5143	2.5702013	0.0188503	0.3511067	-3.791464	MS
LGALS3	-119.8699	249.63665	-2.559146	0.019297	0.3511067	-3.798496	MS
BMPR1A	-68.2464	252.4473	-2.549031	0.0197144	0.3511067	-3.804927	MS
ICOSLG	25.6233	59.77265	2.5451304	0.0198776	0.3511067	-3.807406	MS
PCBP2	-468.1236	3285.2379	-2.541331	0.0200378	0.3511067	-3.80982	MS
TYK2	-162.2467	1152.1117	-2.539541	0.0201137	0.3511067	-3.810957	MS
TCF12	-47.46425	239.35958	-2.524255	0.020773	0.3511067	-3.820663	MS
UBA52	-1108.741	8926.1985	-2.493845	0.0221457	0.3511067	-3.839946	MS
CEBPG	-459.0456	1563.3414	-2.467611	0.0233978	0.3511067	-3.856551	MS
AIM2	-30.3534	54.8485	-2.457645	0.0238907	0.3511067	-3.862851	MS
PRKCD	94.86865	454.31673	2.4392965	0.0248236	0.3511067	-3.874437	MS
BST2	-111.9321	408.83455	-2.432328	0.0251867	0.3511067	-3.878832	MS
FBXO9	19.3158	81.517933	2.4219005	0.0257395	0.3511067	-3.885406	MS
IRAK1BP1	-47.8975	166.18695	-2.418736	0.0259094	0.3511067	-3.887399	MS
IL18	-3419.069	19522.618	-2.416763	0.0260159	0.3511067	-3.888642	MS
TNFRSF6B	-27.5951	97.9549	-2.414358	0.0261463	0.3511067	-3.890157	MS
TLR4	-20.6665	79.03995	-2.410872	0.0263364	0.3511067	-3.892352	MS
CSK	-96.7104	587.0825	-2.409267	0.0264243	0.3511067	-3.893362	MS
TRIM28	-45.6269	340.46535	-2.390529	0.0274714	0.3511067	-3.905146	MS
ITGB1	-141.9398	599.65914	-2.389105	0.0275526	0.3511067	-3.906041	MS
IL10RB	-26.6869	94.12835	-2.385391	0.0277652	0.3511067	-3.908374	MS

TNFRSF10B	-42.9211	104.77015	-2.363945	0.0290234	0.3511067	-3.92183	MS
HMGB1	15.2602	38.7779	2.3607242	0.0292169	0.3511067	-3.923849	MS
SIT1	-8.6416	40.0721	-2.360464	0.0292326	0.3511067	-3.924012	MS
TNFRSF8	-5.4364	17.564	-2.355215	0.0295506	0.3511067	-3.9273	MS
F12	124.9209	549.84665	2.3540529	0.0296215	0.3511067	-3.928028	MS
TGFBR3	-247.6749	660.91865	-2.352011	0.0297463	0.3511067	-3.929306	MS
CFI	-70.4713	271.12575	-2.351607	0.0297711	0.3511067	-3.929559	MS
DEFB118	-5.722	11.0431	-2.348125	0.0299853	0.3511067	-3.931738	MS
BCL2	-67.3579	260.18625	-2.347588	0.0300185	0.3511067	-3.932074	MS
DBNL	252.5	1708.8157	2.3426936	0.0303224	0.3511067	-3.935136	MS
MEF2C	-418.8087	4521.8992	-2.339708	0.0305091	0.3511067	-3.937003	MS
GBP2	-239.797	320.8246	-2.323339	0.0315521	0.3511067	-3.947229	MS
C1R	-32.0619	73.14165	-2.322755	0.0315899	0.3511067	-3.947594	MS
LCP1	-241.0905	436.83955	-2.305565	0.032722	0.3511067	-3.958312	MS
TREM2	-32.9639	59.61295	-2.301185	0.0330164	0.3511067	-3.96104	MS
SIRT1	-103.9929	540.63725	-2.298712	0.0331837	0.3511067	-3.962579	MS
KLRF1	8.0021	22.57665	2.2897002	0.0338	0.3511067	-3.968186	MS
BCL3	-17.1658	55.8223	-2.284117	0.0341871	0.3511067	-3.971656	MS
STAT6	-51.7512	311.684	-2.283658	0.0342191	0.3511067	-3.971941	MS
HLA-DMA	-297.5151	657.61895	-2.278413	0.0345868	0.3511067	-3.975199	MS
NOTCH1	-97.5389	451.85265	-2.273534	0.0349321	0.3511067	-3.978228	MS
LILRA3	-17.0462	19.8195	-2.272126	0.0350324	0.3511067	-3.979102	MS
ITGB2	-547.8949	1177.1853	-2.260077	0.0359011	0.3511067	-3.986572	MS
HLA-DRA	-389.6021	700.88535	-2.257729	0.0360727	0.3511067	-3.988026	MS
PML	-12.82153	63.3205	-2.256066	0.0361947	0.3511067	-3.989056	MS
MAP3K5	-259.026	1231.769	-2.254013	0.0363458	0.3511067	-3.990327	MS
PRKD2	-68.9359	312.40395	-2.251465	0.0365342	0.3511067	-3.991905	MS
SOX9	-454.5427	960.57765	-2.251068	0.0365636	0.3511067	-3.99215	MS
HLA-A	-374.4341	1214.6625	-2.248777	0.0367338	0.3511067	-3.993567	MS
FUT7	-12.9859	33.18685	-2.211987	0.0395697	0.3553202	-4.016271	MS
C3	-178.005	391.1046	-2.203825	0.0402254	0.3553202	-4.021293	MS
TRIM32	86.925	430.8864	2.2031114	0.0402832	0.3553202	-4.021731	MS

**ADSS**	**35.9259**	**167.91735**	**2.2023148**	**0.0403478**	**0.3553202**	**-4.022221**	**MS**
**IFITM1**	**-518.3342**	**1125.5769**	**-2.197927**	**0.0407055**	**0.3553202**	**-4.024917**	**MS**
**GZMH**	**8.0131**	**18.70235**	**2.1906248**	**0.041307**	**0.3553202**	**-4.029401**	**MS**
**COCH**	**97.8087**	**259.21015**	**2.1883142**	**0.0414991**	**0.3553202**	**-4.030818**	**MS**
**HP**	**-13.1348**	**39.375**	**-2.184023**	**0.041858**	**0.3553202**	**-4.03345**	**MS**
**LILRB2**	**10.4977**	**18.46805**	**2.1820136**	**0.0420269**	**0.3553202**	**-4.034682**	**MS**
**CD180**	**11.3566**	**26.9368**	**2.1739588**	**0.0427107**	**0.3553202**	**-4.039616**	**MS**
**TRIM25**	**-11.1332**	**35.3358**	**-2.173762**	**0.0427275**	**0.3553202**	**-4.039736**	**MS**
**LBP**	**-6.785**	**19.84**	**-2.173648**	**0.0427373**	**0.3553202**	**-4.039806**	**MS**
**FOXJ1**	**-42.2922**	**62.5784**	**-2.166097**	**0.0433879**	**0.3553202**	**-4.044426**	**MS**
**CEBPB**	**-994.0571**	**3414.8488**	**-2.165245**	**0.0434619**	**0.3553202**	**-4.044946**	**MS**
**CTSC**	**-25.16675**	**87.277725**	**-2.162725**	**0.0436812**	**0.3553202**	**-4.046486**	**MS**
**HRAS**	**72.2832**	**490.3035**	**2.156763**	**0.0442045**	**0.3553202**	**-4.050128**	**MS**
**SERINC3**	**130.86655**	**1291.6523**	**2.1512723**	**0.0446915**	**0.3553202**	**-4.053479**	**MS**
**RFX1**	**-55.2016**	**403.081**	**-2.148319**	**0.0449553**	**0.3553202**	**-4.05528**	**MS**
**FCER1G**	**-227.1897**	**399.88655**	**-2.141179**	**0.0455993**	**0.3564282**	**-4.059631**	**MS**
**TRAF3IP2**	**-76.49855**	**184.46798**	**-2.135618**	**0.0461067**	**0.3564282**	**-4.063016**	**MS**
**TNFRSF11B**	**-33.9353**	**99.37655**	**-2.132468**	**0.0463964**	**0.3564282**	**-4.064932**	**MS**
**NFKB1**	**-191.7005**	**1177.9552**	**-2.125686**	**0.0470256**	**0.3579174**	**-4.069054**	**MS**
**KLRK1**	**6.9198**	**18.6211**	**2.1083466**	**0.0486699**	**0.3670334**	**-4.079572**	**MS**
**DEFB106A**	**5.9337**	**17.59055**	**2.101862**	**0.0492981**	**0.3677249**	**-4.083497**	**MS**
**POLR3G**	**-9.4018**	**24.1338**	**-2.096361**	**0.0498368**	**0.3677249**	**-4.086824**	**MS**
**NUDCD1**	**30.091833**	**69.79865**	**5.2484176**	**4.87E-05**	**0.0400213**	**-2.515159**	**PD**
**ATG5**	**94.073417**	**414.8032**	**4.776984**	**0.0001378**	**0.0409716**	**-2.712573**	**PD**
**TNFRSF14**	**-439.0969**	**730.656**	**-4.733642**	**0.0001518**	**0.0409716**	**-2.731735**	**PD**
**TRIM28**	**-125.7916**	**363.4902**	**-4.611562**	**0.0001994**	**0.0409716**	**-2.786623**	**PD**
**MCM3AP**	**-186.789**	**784.0048**	**-4.475163**	**0.0002708**	**0.0429129**	**-2.849546**	**PD**
**DENND1B**	**30.714042**	**41.28295**	**4.4025451**	**0.0003188**	**0.0429129**	**-2.883727**	**PD**
**NDFIP1**	**520.11762**	**1465.0063**	**4.2849212**	**0.0004156**	**0.0429129**	**-2.940087**	**PD**
**DRD2**	**159.49842**	**146.1737**	**4.2427488**	**0.0004571**	**0.0429129**	**-2.96059**	**PD**
**IKBKB**	**-189.9115**	**350.2323**	**-4.198587**	**0.0005051**	**0.0429129**	**-2.982225**	**PD**
**SUSD4**	**431.494**	**698.15935**	**4.1601497**	**0.000551**	**0.0429129**	**-3.001192**	**PD**

NOTCH1	-315.1099	697.19605	-4.135444	0.0005826	0.0429129	-3.01345	PD
COL4A3BP	585.66679	1659.7696	4.1033656	0.0006265	0.0429129	-3.029443	PD
GPI	735.44279	2043.5792	4.0003534	0.000791	0.0500124	-3.081382	PD
CSK	-313.8777	1110.5173	-3.944982	0.0008966	0.0518808	-3.109659	PD
IL27RA	-103.1308	149.5921	-3.920949	0.0009467	0.0518808	-3.122009	PD
ARHGEF2	-1963.195	4220.2816	-3.745551	0.0014081	0.0681376	-3.213494	PD
TYK2	-497.0586	1179.0967	-3.745226	0.0014092	0.0681376	-3.213665	PD
SIRT2	-596.6568	1372.3069	-3.650816	0.0017444	0.0796632	-3.26385	PD
TRAFD1	-168.2005	541.8159	-3.579257	0.0020503	0.0887036	-3.302298	PD
IKBKG	-157.233	671.25695	-3.532762	0.002277	0.0935835	-3.32746	PD
RFX1	-139.5908	324.47345	-3.470451	0.00262	0.0971753	-3.361393	PD
ABL1	-119.319	349.5356	-3.458765	0.0026898	0.0971753	-3.367783	PD
SP2	-91.90029	419.8448	-3.453957	0.002719	0.0971753	-3.370414	PD
FGR	-251.5963	398.3428	-3.367801	0.0032995	0.1126398	-3.417789	PD
PVR	102.20796	226.70785	3.3510421	0.0034258	0.1126398	-3.427051	PD
MAP4K2	-480.241	1447.7065	-3.304353	0.0038033	0.1202429	-3.452931	PD
THBS1	241.10092	213.00045	3.2795867	0.0040198	0.1223818	-3.466703	PD
RNF135	-51.43883	181.57905	-3.25274	0.0042682	0.125303	-3.481664	PD
TNFSF10	-111.2405	196.7357	-3.186935	0.0049423	0.1353965	-3.518471	PD
IL5	7.940625	37.16625	3.1860439	0.0049522	0.1353965	-3.518971	PD
TINAGL1	-39.45246	74.03285	-3.17227	0.0051062	0.1353965	-3.526699	PD
SEMA4D	-1165.622	2507.5188	-3.138061	0.0055093	0.136074	-3.545923	PD
ELF4	-42.1845	113.3667	-3.132343	0.0055796	0.136074	-3.54914	PD
IFITM2	-1966.294	5838.8298	-3.127701	0.0056374	0.136074	-3.551753	PD
CMKLR1	-17.99192	46.10115	-3.115346	0.0057939	0.136074	-3.558711	PD
SIAE	270.11879	970.9091	3.0977734	0.0060239	0.1375451	-3.568617	PD
CD55	456.44396	1325.5585	3.0454613	0.0067623	0.1453633	-3.598164	PD
TNFSF12	-61.3346	367.0192	-3.007383	0.0073543	0.1453633	-3.619721	PD
F12	78.107333	118.0471	3.0065864	0.0073672	0.1453633	-3.620173	PD
POU2AF1	-8.351042	30.57225	-3.00157	0.007449	0.1453633	-3.623015	PD
NCF1	-30.19917	49.82625	-2.998776	0.0074949	0.1453633	-3.624599	PD
PSEN1	-32.34994	80.322217	-2.991488	0.007616	0.1453633	-3.628731	PD

NFKB1	-561.586	1642.735	-2.98642	0.0077013	0.1453633	-3.631605	PD
CLEC5A	-47.25246	74.2091	-2.981736	0.007781	0.1453633	-3.634262	PD
TRAF3	23.907333	61.1826	2.9550636	0.0082502	0.1468052	-3.649399	PD
ADAM15	-1031.455	2451.8783	-2.923674	0.0088374	0.1468052	-3.667232	PD
ENPP1	51.345833	119.72375	2.9094978	0.0091157	0.1468052	-3.675292	PD
CCR7	6.1500417	6.93035	2.9088888	0.0091278	0.1468052	-3.675638	PD
LTB4R	-31.94154	63.6753	-2.906953	0.0091665	0.1468052	-3.676739	PD
OSM	17.114667	37.8187	2.9026149	0.0092538	0.1468052	-3.679206	PD
CTLA4	14.394167	80.451	2.8993413	0.0093202	0.1468052	-3.681068	PD
PLCL2	195.84163	694.8319	2.8885903	0.0095415	0.1468052	-3.687184	PD
SAMHD1	-12.91321	35.4793	-2.884328	0.0096307	0.1468052	-3.689609	PD
NCF4	-32.81183	94.90035	-2.883688	0.0096441	0.1468052	-3.689973	PD
CD6	61.150167	90.6564	2.8561957	0.0102395	0.1528447	-3.705621	PD
LILRA2	-146.6019	206.2804	-2.847908	0.0104258	0.1528447	-3.71034	PD
RIPK3	-14.27638	25.67395	-2.840339	0.0105987	0.1528447	-3.71465	PD
TNFAIP1	-161.8625	827.7814	-2.783273	0.0119946	0.1686046	-3.74716	PD
MBP	-1903.835	5920.4034	-2.779157	0.0121018	0.1686046	-3.749506	PD
VNN1	-25.38325	31.0352	-2.766869	0.0124274	0.1686264	-3.756508	PD
STAT6	-50.79796	241.47565	-2.763664	0.0125136	0.1686264	-3.758334	PD
PTK2	-776.6593	3640.4563	-2.724002	0.01363	0.1779568	-3.780935	PD
HIST1H2BK	-816.2986	2160.7236	-2.723694	0.013639	0.1779568	-3.78111	PD
VIPR1	-36.80696	61.4203	-2.710936	0.0140182	0.1800456	-3.788379	PD
IL2	6.725375	29.63565	2.6938205	0.0145426	0.1839077	-3.798129	PD
FCGRT	-269.7871	882.8065	-2.663714	0.0155107	0.1929222	-3.815274	PD
IL6ST	43.62475	135.9884	2.6572977	0.0157248	0.1929222	-3.818927	PD
SFTPD	60.466542	98.48695	2.6450371	0.0161418	0.1951255	-3.825905	PD
UNC93B1	-17.78558	33.08035	-2.631326	0.0166205	0.1980006	-3.833707	PD
CXCL5	10.937792	41.2372	2.6216229	0.0169674	0.1986544	-3.839227	PD
SECTM1	-9.779708	28.83695	-2.616352	0.0171587	0.1986544	-3.842224	PD
LGALS9	-40.02671	72.53615	-2.549995	0.0197487	0.2206739	-3.879917	PD
TNFRSF10D	-16.25054	39.4072	-2.53412	0.0204209	0.2206739	-3.88892	PD
HIST1H2BJ	-21.46479	37.605	-2.529579	0.0206171	0.2206739	-3.891493	PD

DUSP10	-21.54315	73.291575	-2.523088	0.0209007	0.2206739	-3.895172	PD
KIR3DL2	-7.648667	16.22545	-2.521192	0.0209842	0.2206739	-3.896246	PD
TFEB	-31.80271	68.56775	-2.52116	0.0209856	0.2206739	-3.896264	PD
JAM3	-1970.552	7162.5737	-2.518754	0.0210921	0.2206739	-3.897627	PD
ANKHD1	-43.94894	222.68168	-2.516139	0.0212083	0.2206739	-3.899108	PD
GBF1	109.99883	388.36545	2.5019473	0.0218499	0.2233596	-3.907143	PD
POLR3G	68.845667	152.76035	2.4980346	0.02203	0.2233596	-3.909357	PD
TNFSF14	-7.634583	25.29925	-2.492616	0.0222816	0.2233596	-3.912422	PD
DPP8	95.770313	454.23	2.4851182	0.0226343	0.224015	-3.916662	PD
ENPP2	-1980.409	5188.0265	-2.479709	0.022892	0.224015	-3.91972	PD
PTAFR	-40.16617	120.0302	-2.469188	0.0234012	0.225201	-3.925665	PD
FER	42.186	158.39865	2.4659267	0.0235612	0.225201	-3.927507	PD
CSF1R	-1326.76	3360.9365	-2.44108	0.024814	0.23445	-3.941527	PD
IFNA17	-5.43	2.07075	-2.435144	0.0251225	0.2346665	-3.944873	PD
CARD9	-119.7236	222.92215	-2.429327	0.0254281	0.2347383	-3.94815	PD
CD40	-16.76117	51.53995	-2.416508	0.0261142	0.2347383	-3.955368	PD
FUT7	-7.026125	23.0288	-2.413021	0.0263037	0.2347383	-3.95733	PD
HMGB3	93.868542	226.969	2.4129168	0.0263094	0.2347383	-3.957388	PD
PRKD2	-111.0739	365.95435	-2.408382	0.026558	0.2347383	-3.95994	PD
PCBP2	-630.4208	2804.4998	-2.390972	0.0275326	0.2407638	-3.969724	PD
SYK	-91.67713	193.14465	-2.383598	0.0279554	0.2418874	-3.973864	PD
SEMA7A	-7.844458	20.43405	-2.370804	0.0287033	0.2421628	-3.981042	PD
LGALS3	-104.1963	309.7995	-2.359168	0.0293995	0.2421628	-3.987562	PD
SUSD2	-8.807125	37.53465	-2.357656	0.0294912	0.2421628	-3.988409	PD
INPP5D	-244.5539	707.6602	-2.351409	0.0298726	0.2421628	-3.991906	PD
IRF7	-45.02415	163.6923	-2.341639	0.0304783	0.2421628	-3.997372	PD
HRAS	150.95325	480.16705	2.3376703	0.0307275	0.2421628	-3.99959	PD
ICAM2	-537.7632	1199.2302	-2.332026	0.0310853	0.2421628	-4.002744	PD
GGT1	-7.705583	36.222725	-2.331662	0.0311086	0.2421628	-4.002948	PD
TYRO3	-527.8914	1704.2444	-2.326634	0.0314307	0.2421628	-4.005756	PD
IRAK1BP1	58.960958	173.3263	2.3209538	0.0317984	0.2421628	-4.008926	PD
IL13RA2	88.935167	88.6734	2.3179072	0.0319973	0.2421628	-4.010626	PD

PSMB10	690.1905	3630.059	2.3120853	0.0323805	0.2421628	-4.013873	PD
TRIM5	-40.21021	72.794	-2.311595	0.032413	0.2421628	-4.014147	PD
SPPL2B	-18.14229	60.41175	-2.311539	0.0324167	0.2421628	-4.014178	PD
FADD	-75.03229	422.99325	-2.311025	0.0324507	0.2421628	-4.014464	PD
CX3CL1	220.39258	846.0582	2.3064629	0.0327546	0.2421628	-4.017007	PD
FKBP1A	-254.4666	1009.279	-2.302875	0.0329954	0.2421628	-4.019005	PD
FES	-44.20667	144.456	-2.294516	0.0335629	0.2441477	-4.023659	PD
POLR3F	100.81179	320.68455	2.2888938	0.0339496	0.2447944	-4.026787	PD
AZGP1	-528.8398	584.5066	-2.284137	0.03428	0.2450273	-4.029432	PD
OPRD1	13.237625	80.34155	2.2783967	0.0346827	0.2457685	-4.032621	PD
CD4	-53.81604	167.6595	-2.270407	0.0352504	0.2475242	-4.037058	PD
HAMP	1633.2723	1823.8018	2.2664786	0.0355327	0.2475242	-4.039237	PD
HRH2	28.479458	115.6572	2.2485952	0.0368442	0.2545039	-4.049147	PD
SMAD6	-148.2636	285.7843	-2.239212	0.0375501	0.2561035	-4.054338	PD
CD33	-73.978	140.33455	-2.227566	0.0384435	0.2561035	-4.060773	PD
PTK2B	-11.31908	43.422825	-2.223222	0.0387817	0.2561035	-4.063171	PD
TCF12	-163.3943	682.15465	-2.213542	0.0395452	0.2561035	-4.068509	PD
IL6	470.28367	248.77255	2.2118371	0.0396811	0.2561035	-4.069448	PD
IRAK1	-200.1249	1246.7315	-2.209252	0.039888	0.2561035	-4.070872	PD
DOCK2	-193.549	426.56415	-2.202585	0.040426	0.2561035	-4.074543	PD
SERINC3	184.2606	1289.2293	2.2009972	0.0405551	0.2561035	-4.075416	PD
NCF2	-13.41604	55.8255	-2.200445	0.0406002	0.2561035	-4.07572	PD
IFITM1	-508.0223	1459.1669	-2.200161	0.0406233	0.2561035	-4.075876	PD
PML	-12.53394	66.290617	-2.198484	0.0407603	0.2561035	-4.076799	PD
IFITM3	-2146.039	7747.4418	-2.197821	0.0408145	0.2561035	-4.077163	PD
ITGAL	-39.37254	80.35565	-2.182112	0.0421202	0.2622941	-4.085793	PD
ADARB1	186.44165	535.9527	2.1687461	0.0432611	0.2673729	-4.093121	PD
STYK1	24.198917	29.63515	2.1596274	0.0440555	0.2695134	-4.098112	PD
POLR3D	-19.45367	71.12795	-2.154093	0.0445442	0.2695134	-4.101138	PD
LAT	-6.725444	30.05535	-2.153565	0.044591	0.2695134	-4.101426	PD
ICOS	-5.468792	11.33865	-2.147632	0.0451209	0.2707255	-4.104667	PD
IL10RB	-49.86421	160.0844	-2.139991	0.0458118	0.2718756	-4.108836	PD

PTGER4	-95.53233	237.8129	-2.135519	0.0462204	0.2718756	-4.111274	PD
SLPI	-21.88775	47.80465	-2.134601	0.0463049	0.2718756	-4.111775	PD
TRIM11	-35.00913	179.69735	-2.118495	0.0478072	0.2752698	-4.120539	PD
PIK3CD	-54.75346	192.2387	-2.116021	0.0480418	0.2752698	-4.121884	PD
PIK3CG	-75.611	145.85385	-2.114279	0.0482076	0.2752698	-4.12283	PD
OSCAR	-34.40025	53.46415	-2.114124	0.0482224	0.2752698	-4.122914	PD
INPPL1	-249.3235	904.14385	-2.106495	0.0489551	0.2775249	-4.127055	PD
XBP1	141.7203	440.18115	4.8246967	0.0001228	0.1009476	-2.861845	SCHIZ
SAMSN1	46.5196	71.2463	3.6804252	0.001624	0.4490195	-3.364648	SCHIZ
RELB	33.9262	51.8379	3.6734157	0.00165	0.4490195	-3.368033	SCHIZ
CTSC	42.17175	91.938375	3.5491893	0.002185	0.4490195	-3.428538	SCHIZ
ST6GAL1	151.6541	540.7237	3.2320229	0.0044555	0.4657036	-3.586971	SCHIZ
BST1	11.0766	17.6433	3.1863398	0.0049334	0.4657036	-3.610185	SCHIZ
CD300C	15.1086	21.7548	3.1159307	0.0057693	0.4657036	-3.646119	SCHIZ
PTPN6	21.92595	46.749125	3.104545	0.0059169	0.4657036	-3.651947	SCHIZ
TREML4	7.8444	12.4073	3.0296123	0.0069837	0.4657036	-3.690398	SCHIZ
CD274	7.4768	12.6918	2.9631313	0.0080846	0.4657036	-3.72464	SCHIZ
IFI16	185.577	320.6152	2.9401331	0.0085032	0.4657036	-3.736509	SCHIZ
TYROBP	383.4123	547.25955	2.9333087	0.0086314	0.4657036	-3.740033	SCHIZ
CD74	638.2925	995.19205	2.8730292	0.0098476	0.4657036	-3.771195	SCHIZ
IL1RN	8.0439	22.87005	2.8128457	0.0112255	0.4657036	-3.802355	SCHIZ
FOXJ1	-70.6415	78.37495	-2.803112	0.0114651	0.4657036	-3.807398	SCHIZ
TNFSF13B	33.8151	99.89645	2.7713948	0.0122802	0.4657036	-3.823834	SCHIZ
THBS1	75.9612	102.3236	2.7566509	0.0126777	0.4657036	-3.831476	SCHIZ
CD84	12.0434	26.2709	2.7527354	0.0127854	0.4657036	-3.833505	SCHIZ
HIST1H2BK	203.509	932.7534	2.7112788	0.0139797	0.4657036	-3.854993	SCHIZ
HCK	88.5621	150.38075	2.7106466	0.0139987	0.4657036	-3.855321	SCHIZ
CCL2	180.6531	160.07255	2.6834614	0.01484	0.4657036	-3.869409	SCHIZ
KLRF1	10.5619	22.36745	2.6749409	0.0151135	0.4657036	-3.873824	SCHIZ
F2RL1	-11.2517	14.33305	-2.661964	0.0155392	0.4657036	-3.880548	SCHIZ
CYBA	297.6064	408.5342	2.6571394	0.0157003	0.4657036	-3.883047	SCHIZ
DOCK2	106.5776	168.0076	2.6549071	0.0157754	0.4657036	-3.884203	SCHIZ

NCF4	15.3449	63.34135	2.6546028	0.0157857	0.4657036	-3.884361	SCHIZ
APOL1	7.20065	37.032575	2.6350878	0.0164573	0.4657036	-3.894467	SCHIZ
TSC1	-72.4754	340.2992	-2.624264	0.0168414	0.4657036	-3.900071	SCHIZ
LST1	31.53175	70.599625	2.6157242	0.0171505	0.4657036	-3.904491	SCHIZ
PLSCR1	104.2911	154.37775	2.6093743	0.0173837	0.4657036	-3.907778	SCHIZ
LY86	156.0391	245.77515	2.6045488	0.017563	0.4657036	-3.910274	SCHIZ
TREM2	57.383	69.5567	2.563132	0.0191754	0.4681005	-3.93169	SCHIZ
PRKD1	-62.8689	256.17085	-2.561096	0.0192582	0.4681005	-3.932742	SCHIZ
HLA-DPA1	269.7848	460.5108	2.5585576	0.0193618	0.4681005	-3.934053	SCHIZ
BCL2	-74.2504	292.1066	-2.513154	0.0213073	0.4825001	-3.957488	SCHIZ
HRH2	23.7104	90.7926	2.4967944	0.0220522	0.4825001	-3.96592	SCHIZ
HCST	312.1769	522.53765	2.4764892	0.023011	0.4825001	-3.976375	SCHIZ
HIST1H2BI	-7.8646	13.9666	-2.474402	0.0231117	0.4825001	-3.977449	SCHIZ
PTGER4	34.2364	79.9516	2.4581426	0.0239108	0.4825001	-3.985812	SCHIZ
IRAK1	228.7053	1096.2789	2.4541845	0.0241092	0.4825001	-3.987846	SCHIZ
NCF2	16.8218	44.121	2.4141214	0.0262059	0.4825001	-4.008409	SCHIZ
BMP6	69.4845	169.17685	2.4121641	0.0263126	0.4825001	-4.009412	SCHIZ
ADAR	-257.4266	2183.9495	-2.40081	0.0269395	0.4825001	-4.015228	SCHIZ
POLR3G	21.9831	59.50745	2.3873455	0.027701	0.4825001	-4.022119	SCHIZ
LCP2	66.6205	203.67145	2.375936	0.0283619	0.4825001	-4.027952	SCHIZ
CST7	21.1009	75.10735	2.3735386	0.0285026	0.4825001	-4.029177	SCHIZ
IL1RAP	17.0572	49.4638	2.3731297	0.0285267	0.4825001	-4.029386	SCHIZ
CYBB	78.6884	137.4924	2.3502224	0.0299056	0.4825001	-4.041078	SCHIZ
IL32	23.3767	24.96255	2.3366297	0.0307528	0.4825001	-4.048005	SCHIZ
CD55	201.458	676.6628	2.3339709	0.0309211	0.4825001	-4.049359	SCHIZ
FYB	58.7322	94.46685	2.3332883	0.0309644	0.4825001	-4.049706	SCHIZ
LCP1	268.3375	383.92075	2.3272287	0.0313517	0.4825001	-4.05279	SCHIZ
C2	26.7948	42.9716	2.3255516	0.0314597	0.4825001	-4.053644	SCHIZ
NMI	93.5751	247.86965	2.2829541	0.0343204	0.4825001	-4.075269	SCHIZ
CCR1	29.9088	46.4992	2.2803513	0.0345028	0.4825001	-4.076587	SCHIZ
KIR2DS4	-10.3285	41.74155	-2.266756	0.0354699	0.4825001	-4.083466	SCHIZ
IL1A	22.1476	33.5584	2.2664902	0.0354891	0.4825001	-4.083601	SCHIZ

LYN	200.1895	510.03795	2.2567294	0.0361992	0.4825001	-4.088534	SCHIZ
OPRD1	-14.6891	87.62165	-2.233157	0.0379684	0.4825001	-4.100423	SCHIZ
SLC11A1	108.25535	104.10543	2.2278924	0.0383742	0.4825001	-4.103074	SCHIZ
ELF4	18.5188	51.5365	2.1926105	0.0411984	0.4825001	-4.120793	SCHIZ
HLA-DQB1	-33.5614	44.8763	-2.18841	0.041547	0.4825001	-4.122896	SCHIZ
LBP	11.3436	20.1186	2.1841556	0.0419029	0.4825001	-4.125027	SCHIZ
FASLG	6.4238	13.651	2.1766515	0.0425374	0.4825001	-4.12878	SCHIZ
HLA-DRA	425.9722	555.9211	2.1718199	0.0429505	0.4825001	-4.131195	SCHIZ
TRIM14	-11.3614	47.1253	-2.16719	0.0433498	0.4825001	-4.133508	SCHIZ
HLA-DMA	350.0759	604.16295	2.1660123	0.043452	0.4825001	-4.134096	SCHIZ
GBP2	284.7664	364.2203	2.161295	0.0438632	0.4825001	-4.13645	SCHIZ
HLA-B	595.475	1092.7402	2.1568865	0.0442507	0.4825001	-4.138648	SCHIZ
MEF2C	-742.6315	4355.6855	-2.146841	0.0451454	0.4825001	-4.143653	SCHIZ
SELL	19.6056	58.8841	2.1451573	0.045297	0.4825001	-4.144491	SCHIZ
CD1D	16.9958	74.9345	2.1421904	0.0455653	0.4825001	-4.145968	SCHIZ
LY96	292.3658	419.1776	2.1414014	0.0456368	0.4825001	-4.14636	SCHIZ
RPS27A	1948.2942	7534.2524	2.1349161	0.046229	0.4825001	-4.149585	SCHIZ
ALCAM	518.5663	2414.1098	2.1329127	0.0464134	0.4825001	-4.15058	SCHIZ
BTK	56.0077	116.71775	2.1307102	0.0466168	0.4825001	-4.151674	SCHIZ
HLA-E	1355.2584	2234.6617	2.1306779	0.0466198	0.4825001	-4.15169	SCHIZ
MYD88	33.5718	87.3201	2.1282332	0.0468465	0.4825001	-4.152904	SCHIZ
CD209	18.6441	19.91595	2.1272442	0.0469386	0.4825001	-4.153395	SCHIZ
LILRA1	18.1532	72.689	2.1270285	0.0469586	0.4825001	-4.153502	SCHIZ
HAMP	1024.3303	927.38265	2.112934	0.0482886	0.4854935	-4.16049	SCHIZ
HLA-DOA	34.8756	58.8129	2.1112883	0.0484461	0.4854935	-4.161305	SCHIZ
APOBEC3D	-9.197	23.9985	-2.105313	0.0490218	0.4854935	-4.164262	SCHIZ

S3 Fig. FDR vs. -log (*p*-value). The changes in FDR (BF-corrected p-value) versus -log (p-value) for the first 300000 results of fastLA. As shown, FDR = 0.01 corresponds to -log (p- value) = 7.30.

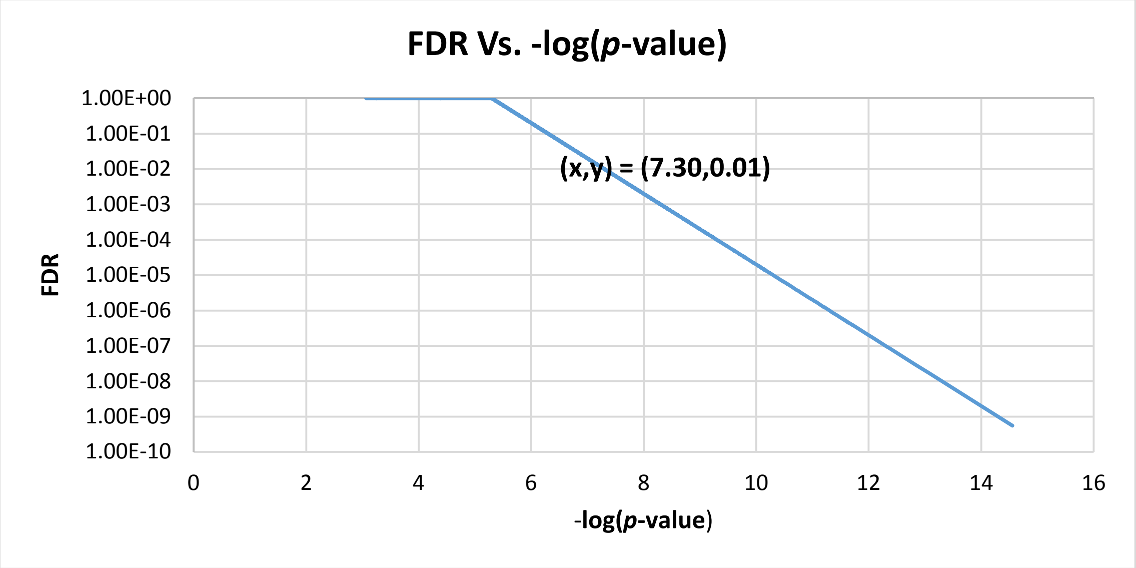

S4 Table. KEGG pathway enrichment analysis information.

GOTerm	Disease	FDR	(-log FDR)
Cytokine-cytokine receptor interaction	ALS	6.03E-08	7.22
	MS	1.50E-03	2.82
	HD	1.74E-03	2.76
	SCHIZ	3.67E-03	2.44
	PD	6.06E-03	2.22
	AD	2.58E-02	1.59
Natural killer cell mediated cytotoxicity	HD	1.38E-07	6.86
	ALS	2.32E-07	6.63
	PD	1.65E-06	5.78
	MS	2.10E-05	4.68
	SCHIZ	2.57E-03	2.59
Osteoclast differentiation	HD	4.83E-14	13.32
	PD	8.14E-13	12.09
	ALS	3.54E-10	9.45
	MS	7.67E-09	8.12
	SCHIZ	2.06E-07	6.69
B cell receptor signaling pathway	HD	1.68E-08	7.77
	PD	6.07E-07	6.22
	ALS	1.14E-06	5.94
	SCHIZ	2.84E-02	1.55
Cytosolic DNA-sensing pathway	HD	8.69E-13	12.06

	ALS	2.21E-06	5.66
	SCHIZ	2.68E-03	2.57
	AD	5.59E-03	2.25
Epstein-Barr virus infection	HD	1.15E-11	10.94
	ALS	1.10E-09	8.96
	SCHIZ	1.14E-05	4.94
	AD	7.62E-03	2.12
Leukocyte transendothelial migration	SCHIZ	8.86E-04	3.05
	ALS	1.43E-03	2.84
	HD	8.51E-03	2.07
	PD	1.95E-02	1.71
NF-kappa B signaling pathway	HD	1.87E-12	11.73
	ALS	6.16E-10	9.21
	SCHIZ	2.31E-06	5.64
	AD	2.22E-02	1.65
Chemokine signaling pathway	HD	7.46E-06	5.13
	MS	3.90E-03	2.41
	SCHIZ	7.44E-03	2.13
Neurotrophin signaling pathway	PD	3.27E-04	3.49
	HD	6.68E-04	3.18
	MS	9.07E-04	3.04
Pathogenic Escherichia coli infection	PD	6.18E-03	2.21
	MS	8.57E-03	2.07
	HD	9.13E-03	2.04
Phagosome	PD	2.40E-10	9.62
	SCHIZ	3.29E-09	8.48
	ALS	6.98E-09	8.16
Primary immunodeficiency	HD	4.54E-03	2.34
	MS	6.38E-03	2.20
	PD	2.38E-02	1.62
NOD-like receptor signaling pathway	PD	4.46E-11	10.35
	ALS	1.59E-06	5.80
	SCHIZ	3.45E-05	4.46
Fc gamma R-mediated phagocytosis	PD	2.58E-03	2.59
	HD	4.39E-03	2.36
Leishmaniasis	MS	4.35E-09	8.36
	SCHIZ	8.47E-09	8.07
Legionellosis	PD	6.18E-03	2.21
	SCHIZ	4.17E-02	1.38
Tuberculosis	PD	2.17E-11	10.66
	MS	1.18E-10	9.93
Cell adhesion molecules (CAMs)	AD	1.25E-02	1.90
Malaria		3.27E-02	1.48
Apoptosis	ALS	1.71E-03	2.77
Herpes simplex infection		2.15E-07	6.67

Intestinal immune network for IgA production		1.11E-03	2.95
Rheumatoid arthritis		2.27E-07	6.64
Staphylococcus aureus infection		3.99E-04	3.40
AGE-RAGE signaling pathway in diabetic complications	HD	3.36E-02	1.47
Prion diseases		3.29E-03	2.48
Shigellosis		1.03E-03	2.99
Viral myocarditis		3.83E-11	10.42
Viral carcinogenesis		5.34E-03	2.27
Antigen processing and presentation	MS	8.66E-10	9.06
Pertussis		7.77E-07	6.11

S5 Table. Biologically relevant triplets. By detecting statistically significant triplets in the enriched KEGG terms, 89 triplets in which X1 and X2 are involved in the same pathway were determined.

Trip Num	X1 or X2	X2 or X1	X3	rhodiff	MLA value	wald	p value	bonferroni
1	IFI16	IKBKB	DRD2	-0.72	-0.33	62.44	2.77556E-15	5.56E-10
19	IFI16	TYK2	DRD2	-1.06	-0.41	47.94	4.3946E-12	0.000000878
29	HLA-DPA1	IKBKB	DRD2	-0.79	-0.37	46.2	1.06777E-11	0.00000214
56	CORO1A	CYBA	IKBKE	1.348	0.4953	43.4714	4.30201E-11	0.0000086
114	LTBR	IKBKB	DRD2	-0.86	-0.32	40.2	2.29248E-10	0.0000458
130	TYK2	HLA-DRA	DRD2	-1.06	-0.45	39.56	3.18133E-10	0.0000636
147	HLA-B	TYK2	DRD2	-1.03	-0.38	39	4.23806E-10	0.0000848
206	TRAF3	CYBA	TREM2	0.5591	0.343	37.7376	8.09295E-10	0.0001618
225	BTK	CD81	DRD2	-0.93	-0.38	37.23	1.04986E-09	0.00021
230	TYROBP	TYK2	DRD2	-1.07	-0.41	37.18	1.07713E-09	0.000216
256	CXCL14	TNFRSF14	ATP6V0A2	-1.1449	-0.4201	36.7916	1.31455E-09	0.000262
268	ITGAL	TYK2	DRD2	-1.02	-0.39	36.63	1.42817E-09	0.000286
269	HLA-DOA	TYK2	DRD2	-1.08	-0.44	36.63	1.42817E-09	0.000286
279	ITGB1	TLR4	ENPP2	-0.7234	-0.3698	36.5234	1.50844E-09	0.000302
304	LTBR	BCL2	IL18	-0.91	-0.34	36.15	1.82698E-09	0.000366
341	HLA-B	HIST2H2BE	SERINC3	0.8051	0.3272	35.6572	2.35277E-09	0.00047
345	TYK2	HLA-A	DRD2	-0.98	-0.41	35.57	2.46048E-09	0.000492
379	LYN	IKBKB	DRD2	-0.87	-0.32	35.13	3.08413E-	0.000616

							09	
381	ITGB2	CORO1A	OSCAR	0.9347	0.3872	35.0669	3.1857E-09	0.000638
384	HLA-DOA	IKBKB	IL13RA2	-0.8578	-0.3372	35.0139	3.2736E-09	0.000654
406	C1QC	NOTCH1	DRD2	-1.08	-0.41	34.75	3.74879E-09	0.00075
409	HLA-DOA	IKBKB	DRD2	-0.76	-0.38	34.73	3.7875E-09	0.000758
452	TYK2	SYK	DRD2	-1.06	-0.42	34.4	4.48723E-09	0.000898
525	CXCL14	TNFRSF14	C1QBP	1.3	0.52	33.94	5.68381E-09	0.001136
541	CXCL14	TNFRSF14	PRKD2	-1.2514	-0.4652	33.8258	6.02742E-09	0.001206
545	C1QA	NOTCH1	DRD2	-0.95	-0.39	33.8	6.10789E-09	0.001222
613	PRKCD	CAMK4	ANG	-0.859	-0.3296	33.4013	7.49738E-09	0.0015
623	LTBR	BCL2	EIF2AK4	-0.7265	-0.3146	33.3403	7.73627E-09	0.001548
652	TYK2	CAMK4	IL27RA	-0.9	-0.38	33.16	8.48787E-09	0.001698
662	CYBB	CORO1A	TLR8	0.9544	0.3324	33.1205	8.66206E-09	0.001732
665	HLA-E	IKBKE	CORO1A	0.98	0.35	33.11	8.70897E-09	0.001742
676	SRC	LTBR	FBXO9	0.8665	0.3838	33.0401	9.02776E-09	0.001806
701	CORO1A	CYBA	TLR8	1.1091	0.3927	32.9308	9.54982E-09	0.00191
721	TREM2	TYK2	DRD2	-1.19	-0.44	32.81	1.01621E-08	0.00204
738	TYK2	IRAK4	DRD2	-0.95	-0.35	32.72	1.06436E-08	0.00212
740	CORO1A	CYBA	FCGR1A	1.2471	0.4479	32.707	1.07151E-08	0.00214
785	TICAM1	IL18	PTGER4	1.1071	0.4155	32.4436	1.22702E-08	0.00246
793	CORO1A	CYBA	SELL	1.03	0.45	32.39	1.26134E-08	0.00252
806	CXCL14	TNFRSF14	PTK2	-1.1865	-0.4886	32.3601	1.2809E-08	0.00256
832	CORO1A	CYBA	BCL3	1.234	0.4596	32.2407	1.36207E-08	0.00272
880	TYK2	OSCAR	DRD2	-1.1	-0.4	32.04	1.51031E-08	0.00302
923	CXCL14	TNFRSF21	POLR3K	1.0021	0.3628	31.8946	1.62769E-08	0.00326
944	LY96	BCL2	NFKB1	-0.7563	-0.3167	31.8023	1.7069E-08	0.00342
950	PTK2B	IKBKG	IL18	-1.22	-0.43	31.78	1.72662E-08	0.00346
952	TICAM1	IL18	VIPR1	-1.17	-0.41	31.78	1.72662E-08	0.00346
956	HLA-B	HIST2H2BE	PRKD2	-0.9173	-0.32	31.7576	1.74664E-08	0.0035

979	IRAK1	IKBKB	IRAK3	1.1729	0.4576	31.6598	1.83685E-08	0.00368
980	PRKCD	IKBKE	SERINC3	-0.9575	-0.3864	31.6527	1.84358E-08	0.00368
996	ITGB1	CORO1A	IKBKE	1.154	0.4062	31.6032	1.89118E-08	0.00378
1005	HLA-DRA	IKBKB	DRD2	-0.7	-0.37	31.55	1.9437E-08	0.00388
1018	ITGB1	IRAK1	IRAK3	1.0649	0.3831	31.5235	1.97041E-08	0.00394
1056	TRAF3	HLA-A	TNFAIP1	-1.0988	-0.3952	31.3909	2.10967E-08	0.00422
1057	TLR2	CORO1A	CACNA1C	-1.03	-0.37	31.39	2.11065E-08	0.00422
1063	CORO1A	CYBA	CD300C	1.18	0.41	31.37	2.13251E-08	0.00426
1064	TICAM1	IL18	IRAK4	1.0529	0.4306	31.3617	2.14164E-08	0.00428
1078	IRAK1	TYK2	IRAK3	1.1505	0.465	31.3181	2.19028E-08	0.00438
1105	CORO1A	CYBA	RNASE3	1.24	0.41	31.22	2.3038E-08	0.0046
1109	TNFRSF1A	CORO1A	IKBKE	1.2364	0.4528	31.2155	2.30915E-08	0.00462
1133	IL18	CX3CL1	RPS27A	0.9307	0.3281	31.1452	2.39431E-08	0.00478
1157	ABL1	HLA-F	TRAF3	0.8712	0.3328	31.0813	2.47444E-08	0.00494
1193	HLA-B	HIST2H2BE	CD81	-0.85	-0.34	30.98	2.60701E-08	0.00522
1218	CXCL14	TNFRSF14	EIF2AK4	-1.227	-0.4382	30.8921	2.7278E-08	0.00546
1224	CHUK	TNFRSF11B	TAPBP	1.0368	0.3472	30.874	2.75336E-08	0.0055
1228	C3	CORO1A	FCGR1A	1.2123	0.4128	30.8595	2.77401E-08	0.00554
1231	C1QB	NOTCH1	DRD2	-0.96	-0.38	30.85	2.78762E-08	0.00558
1234	PTK2	CXCL14	TNFRSF14	-1.003	-0.3464	30.843	2.79769E-08	0.0056
1237	SH2D1B	SYK	DRD2	0.54	0.33	30.84	2.80202E-08	0.0056
1262	TLR4	ABL1	LTBR	-0.85	-0.3	30.77	2.90494E-08	0.0058
1265	ICAM1	FYN	POLR3H	-0.9932	-0.3791	30.7635	2.91469E-08	0.00582
1281	HLA-E	CORO1A	IKBKE	1.1794	0.4335	30.7109	2.99478E-08	0.00598
1298	HLA-DPA1	TYK2	APP	1.0396	0.3736	30.6765	3.04835E-08	0.0061
1306	TICAM1	C1QBP	PLSCR1	-0.9891	-0.3859	30.6618	3.07153E-08	0.00614
1328	LTBR	IL18	BCL2	-1.0241	-0.3664	30.6227	3.13405E-08	0.00626
1335	TLR4	CORO1A	HLA-F	1.19	0.41	30.6	3.17094E-08	0.00634
1336	TLR4	CORO1A	HLA-F	1.19	0.41	30.6	3.17094E-08	0.00634

1345	NFKB1	CORO1A	IKBKE	1.2289	0.4691	30.5762	3.21007E-08	0.00642
1376	HLA-E	HIST2H2BE	TRIM21	-0.9059	-0.3371	30.5068	3.32698E-08	0.00666
1403	ITGB2	TNFSF13B	TRAFD1	0.7662	0.332	30.4343	3.45367E-08	0.0069
1405	HSPD1	CORO1A	NCF2	1	0.36	30.43	3.46134E-08	0.00692
1424	FYN	TNFRSF10B	UBB	1.2	0.44	30.39	3.53345E-08	0.00706
1430	HIST2H2BE	HLA-A	CD81	-0.84	-0.35	30.38	3.55171E-08	0.0071
1454	ITGB2	SFTPD	LCP2	-0.84	-0.34	30.31	3.68223E-08	0.00736
1499	CYBB	TYK2	DRD2	-1.11	-0.42	30.21	3.87705E-08	0.00776
1549	CD14	CORO1A	IFITM2	0.9512	0.3804	30.0689	4.16965E-08	0.00834
1560	HIST2H2BE	IRF7	F12	1.014	0.3683	30.0471	4.21679E-08	0.00844
1565	CCL21	IL18	PRKCE	1.2293	0.4636	30.0325	4.24865E-08	0.0085
1578	TREM2	IKBKB	DRD2	-0.83	-0.35	30	4.32046E-08	0.00864
1590	PRKCD	CXCL14	TAPBP	-0.963	-0.3334	29.9667	4.3953E-08	0.0088
1693	TICAM1	C1QBP	IL1A	-1.0278	-0.3699	29.752	4.90999E-08	0.00982
